# Bioprospecting of Ribosomally Synthesized and Post-translationally Modified Peptides Through Genome Characterization of a Novel Probiotic *Lactiplantibacillus plantarum* UTNGt21A Strain: A Promising Natural Antimicrobials Factory

**DOI:** 10.3389/fmicb.2022.868025

**Published:** 2022-04-06

**Authors:** Gabriela N. Tenea, Pamela Ascanta

**Affiliations:** Biofood and Nutraceutics Research and Development Group, Faculty of Engineering in Agricultural and Environmental Sciences, Technical University of the North, Ibarra, Ecuador

**Keywords:** *Lactiplantibacillus plantarum*, RiPP-like peptides, *de novo* sequencing, bacteriocins, genome mining tools

## Abstract

The present work describes the genome sequencing and characterization of a novel *Lactiplantibacillus plantarum* strain assigned UTNGt21A isolated from wild *Solanum quitoense* (L.) fruits. *In silico* analysis has led to identifying a wide range of biosynthetic gene clusters (BGCs) and metabolic compounds. The genome had a total of 3,558,611 bp with GC of 43.96%, harboring 3,449 protein-coding genes, among which 3,209 were assigned by the EggNOG database, and 240 hypothetical proteins have no match in the BLASTN database. It also contains 68 tRNAs, 1 23S rRNA, 1 16S rRNA, 6 5S rRNA, and 1 tmRNA. In addition, no acquired resistance genes nor virulence and pathogenic factors were predicted, indicating that UTNGt21A is a safe strain. Three areas of interest (AOI) consisting of multiple genes encoding for bacteriocins and ABC transporters were predicted with BAGEL4, while eight secondary metabolite regions were predicted with the antiSMASH web tool. GutSMASH analysis predicted one metabolic gene cluster (MGC) type pyruvate to acetate-formate, a primary metabolite region essential for anaerobe growth. Several lanthipeptides and non-ribosomal peptide synthetase (NRPS) clusters were detected in the UTNGt21A but not the reference genomes, suggesting that their genome diversity might be linked to its niche-specific lineage and adaptation to a specific environment. Moreover, the application of a targeted genome mining tool (RiPPMiner) uncovered a diverse arsenal of important antimicrobial molecules such as lanthipeptides. Furthermore, *in vitro* analysis indicated that the crude extract (CE) of UTNGt21A exerted a wide spectrum of inhibition against several pathogens. The results indicated that the possible peptide-protein extract (PC) from UTNGt21A induces morphological and ultrastructural changes of *Salmonella enterica* subsp. *enterica* ATCC51741, compatible with its inhibitory potential. Genome characterization is the basis for further *in vitro* and *in vivo* studies to explore their use as antimicrobial producers or probiotic strains.

## Introduction

An extremely versatile species, *Lactiplantibacillus plantarum*, conventionally *Lactobacillus plantarum*, generally recognized as safe (GRAS) by the U.S. Food and Drug Administration and qualified as the presumption of safety status by the European Food Safety Authority (EFSA), is investigated for its use to produce diverse probiotics foods (Bernardeau et al., 2006; Leuschner et al., 2010; Gao et al., 2020; Goel et al., 2020). There is a continuous interest in the characterization of *L. plantarum* species as they produce a diverse range of beneficial metabolites such as bacteriocins that might increase food quality by increasing their shelf life (Huan et al., 2020). They produce flavors and enhance the production of active biomolecules, however, providing overall health benefits (Rodrigo-Torres et al., 2019). Besides, the modified bacteriocins such as lanthipeptides, cyclized peptides, sactipeptides, linear azol(in)e containing peptides or lasso peptides, and glycosylated bacteriocins, are considered as the next generation of antibiotics (Ongey and Neubauer, 2016). As well, several strains are producing a large group of natural substances such as RiPP-like molecules (ribosomally synthesized and post-translationally modified peptides), PKs (polyketides), NRPs (non-ribosomally produced peptides), terpenes with application in medicine (Montalbán-López et al., 2021). Considering these numerous attributes along with the strains’ flexibility to adapt in several environments make *L. plantarum* strains an ideal and effective probiotic or antimicrobials producer which might confer positive health benefits to humans (Fidanza et al., 2021; Rocchetti et al., 2021).

Currently (February 2022), 671 genome assembly and annotation reports of *L. plantarum* strains are registered in the NCBI database, showing high genome variability, with *L. plantarum* WCFS1 being the most studied reference strain (Kleerebezem et al., 2003; van den Nieuwboer et al., 2016). However, some strains were found stress-tolerant, adaptable to the dairy matrix, and showing commercial potential (Gao et al., 2020). *In silico* analysis of whole genome sequencing and the use of genome mining tools [a computational method for the automatic detection and annotation of biosynthetic gene clusters (BGCs) from genomic data] has led to a wide comprehension of the beneficial properties, including their genes, safety, and metabolic features of several lactic bacteria strains (Chen et al., 2019; Russell and Truman, 2020). According to recent research, these tools revealed a substantial repository of earlier undetected BGCs in bacteria, highlighting their potential as producers of natural active molecules (Kloosterman et al., 2020).

Most probiotic commercial strains are of human origin, nonetheless, there is sufficient evidence demonstrating the probiotic features of lactobacilli from fermented fruits and vegetables (Swain et al., 2014). Nowadays, several fermented foods known as functional foods containing a consortium of probiotic cultures are getting more impact among the consumers, indicating the high tendency of the probiotic market to shift from dairy to vegetable fermented products (Behera et al., 2018). Various fermented foods such as *pickles, sauerkraut*, Korean *kimchi, brined olives, sourdough*, Nigerian *ogi* containing *L. plantarum* cultures are consumed and they are considered as valuable food systems due to their both nutritional and health benefits (Swain et al., 2014; Park et al., 2016). Thus, the positive perceptions of microbes associated with food raw materials along with their beneficially impact on host health offer a new perspective to develop functional products with a great impact on life quality (Fidanza et al., 2021). In a recent review report from 40 different probiotic species and 42 different delivery technologies, the global probiotic supplements market was predicted to grow by $65 billion in 2024 (Hudson and Daniellls, 2020). However, the probiotic “space” is providing unique opportunities for suppliers and manufacturers looking to grow their share of the market. Besides, the probiotic “inventory” should include microorganisms isolated from unconventional sources as they showed similar or improved characteristics to the conventional strains.

With the aiming of selecting a novel, lactobacilli strain with antimicrobial capacity or probiotic potential, we previously prospected the wild *Solanum quitoense* L. shrub (commonly known as naranjilla) fruits for the presence of lactic bacteria (Tenea et al., 2020). These fruits appear to be a valuable source of beneficial microorganisms with biotechnological features that might be inherited from their ecological origin. Among several selected colonies presumed as lactic acid bacteria (LAB), the isolate assigned UTNGt21A has been resequenced to identify gene variants and discern metabolic features and genes linked to the adaptability of bacteria to different environments (Tenea, 2022). In this study, de novo genome sequencing of UTNGt21A isolate was performed for species classification, gene prediction, and functional annotation, giving new insights into the biology of this species. Its whole genome was analyzed for an evolutionary relationship with other LAB. In addition, we used several computational genome mining tools for the prediction of bacteriocin cluster gene organization, RiPP-like gene clusters, and other primary and secondary metabolites. The strain was also evaluated for safety issues. Besides, the inhibitory spectrum was evaluated with the crude extract (CE) from the UTNGt21A strain against several food pathogens. Moreover, the effect of peptide-protein extract (PC) from UTNGt21A strain on the morphological and ultrastructural bacterial cell changes was evaluated *via* transmission electronic microscopy (TEM) and scanning electronic microscopy (SEM). *In silico* prediction and prospection of the molecules from UTNGt21A genome analysis will further help in improving our knowledge of the utility of *L. plantarum* strains originated from tropical fruits as a promising alternative for novel antimicrobials production or to explore their potential as a probiotic strain. Thus, the characterization of novel *L. plantarum* strains using both *in silico* and *in vitro* approaches will facilitate standardization of research applications and allow for the more rapid and efficient validation of specific strains for defined health utilization.

## Materials and Methods

### Bacterial Strains

In a previous study, several LAB from native *S. quitoense* (Lam.) wild fruits were selected based on their capacity to inhibit several pathogens isolated from local foods (Tenea et al., 2020). Among several cocci- and rod-shaped bacteria, the isolate with the code UTNGt21A was selected for further taxonomical identification and genome characterization. Genome assembly data of UTNGt21A were deposited in the NCBI database: accession PRIJNA740042 and BioSample SAMN19816459 (22 June 2021) (Tenea, 2022). The assembly of *L. plantarum* WCFS1 (GCF_000203855.3) and *L. plantarum* ATCC8014 (GCF_002749655.1), were used as a reference for mapping genome and bacteriocin gene cluster comparison, respectively.

### *De novo* Whole-Genome Sequencing

*De novo* whole-genome sequencing and assembly were performed as previously described (custom service, Macrogen Inc., Seoul, South Korea) (Tenea et al., 2020). In brief, random fragmentation of the DNA or cDNA sample, followed by 5′ and 3′ adapter ligation was used to prepare the sequencing library using a protocol developed by the manufacturer. Subsequently, the raw data were tested for quality (FastQC v0.11.5^1^). The Trimmomatic v0.36 was used to remove the adapter sequences (Bolger et al., 2014). The quality of filtered reads, total base, total reads, GC content, and basic statistics were determined as described (Tenea et al., 2020). *De novo* assembly was performed by various *k*-mer using SPAdes 3.15.1 (Bankevich et al., 2012). *K*-mer analysis provides information about coverage, heterozygosity, and estimated genome size (Marcais and Kingsford, 2011). Using filtered reads, *de novo* assembly was performed using a De Bruijn graph assembler (Vurture et al., 2017). However, 18,074,974 reads were produced, and the total read bases are 1.8G bp. The GC content was 43.31% and Q30 was 85.51%. The genome validation by mapping strategy and BUSCO analysis were performed as described (Tenea et al., 2020). A total of 38 contigs were assembled with total contig bases of 3,558,611, with an N50 value of 188,693. A summary of the assembly results, the mapping overall results, and BUSCO analysis are depicted in Supplementary Tables 1–3. After a complete genome or draft genome was assembled, BLAST analysis was carried out to identify to which species each scaffold shows similarity. Best hit results were identified using the NCBI NT database. The ANI (Average Nucleotide Identity) values of ≥95–96% were used as a criterion to confirm the species, by comparison with the selected reference sequences, *L. plantarum* WCFS1 and *L. plantarum* UTNGt2 (Richter and Rossello-Mora, 2009). A circular map was generated using the web program CGview server (Bristo et al., 2016). Moreover, the genome sequence data were uploaded to the Type (Strain) Genome Server (TYGS), a free bioinformatics platform for a whole genome-based taxonomic analysis (Meier-Kolthoff and Göker, 2019).

### Gene Prediction and Functional Annotation

The UTNGt21A genome was structurally annotated using the PROKKA suite (Seemann, 2014). The CDS, rRNA, tRNA/tmRNA, signal leader peptide, and the non-coding RNA prediction were performed using the Prodigal (Hyatt et al., 2010), RNAmmer (Lagesen et al., 2007), Aragorn (Laslett and Canback, 2004), Signal IP (Nielsen et al., 2019), and Infernal (Nawrocki and Eddy, 2013). The InterProScan (Blum et al., 2021) and EggNOG DB (Huerta-Cepas et al., 2019) were used for functional annotation.

### Prediction of CRISPR Sequences, Prophage, and Mobile Elements

CRISPRFinder (Grissa et al., 2007) and PHAge Search Tool Enhanced Release (PHASTER) (Arndt et al., 2016) were used to detect, CRISPR, Cas sequences, truncated Cas sequences, identification, and annotation of prophage sequences. Plasmid Finder 2.0 was used to search for mobile replicons from assembled genome contigs with 95% minimum identity and 60% of sequence coverage (Carattoli et al., 2014).

### Antibiotic-Resistant Genes, Putative Virulence Gene Prediction, and Pathogenicity Prediction

The Comprehensive Antibiotic Resistance Database (CARD) (Jia et al., 2017) and the Resistance Gene Identifier tool (RGI) were used to detect the antibiotic resistance genes by importing the contig files in FASTA format to the database under Perfect and Rigorous hit and High-quality coverage criteria (Zankari et al., 2012). The ResFinder 4.1 server was used to identify acquired antimicrobial resistance genes with a selected% ID threshold of 90.00% and the selected minimum length of 60% and/or chromosomal mutations (Bortolaia et al., 2020). The putative virulence factors were predicted using the VFDB webserver (Liu et al., 2019). The bacterial pathogenicity was predicted using the PathogenFinder webserver (Cosentino et al., 2013).

### Prediction of Bacteriocins, Primary and Secondary Metabolites, and RiPP-Clusters

The detection of BGCs of antimicrobial compounds was investigated using the BAGEL4 web server (de Jong et al., 2006). For the detection of primary and secondary metabolites from anaerobe bacteria, the input FASTA contig of UTNGt21A was imported in both gutSMASH (Specialized Primary Metabolite Analysis from Anaerobic Bacteria) (Pascal Andreu et al., 2021) and antiSMASH version 6.0.1 (Antibiotic and Secondary Metabolites Shell) (Blin et al., 2021). GutSMASH predicts about 41 primary metabolite pathways, including genes involved in bioenergetics, while antiSMASH allows for the prediction of PKs, NRPs, RiPP-like peptides, terpenes, covering a wide range of known or putative secondary metabolite compounds. An overview of the detected regions in the contigs is displayed (Blin et al., 2019). In addition, using the RiPPMiner-Genome web server, the BGCs for RiPPs were predicted using as input the UTNGt21A contig sequences in FASTA format. Subsequently, the depiction of the chemical structure of crosslinked RiPP was determined (Agrawal et al., 2021).

### Screening of Inhibitory Spectrum of Crude Extract of UTNGt21A Strain Against Several Food Pathogens

The CE was obtained from overnight growth culture of UTNGt21A strain and recovered by centrifugation at 13,000 × *g* for 20 min (4°C) followed by filtration using 0.22 μm porosity syringe filter (# STF020025H, ChemLab Group, United States) and used in agar-well diffusion analysis. To rule out the possible inhibition activity of organic acids, the CE was heated at 80°C for 10 min and the pH was adjusted at 6.0. The following strains were used as indicators: *Staphylococcus aureus* ATCC1026, *S. aureus* ATCC43300, *Listeria monocytogenes* ATCC19115, *Shigella sonnei* ATCC25931, *S. enterica* subsp. *enterica* ATCC51741, *Escherichia coli* ATCC25922, *Enterobacter hormaechei* UTNB3Sh1(a laboratory strain isolated from commercial tropical juice), *Kosakonia cowanii* UTNB2Sh1 (a laboratory strain isolated from commercial orange juice), and *Shigella diphtheriae* UTNFa37-1 (a laboratory strain isolated from local cheese). In brief, the indicator strains (100 μL) grown in appropriate medium (7 log CFU/mL) were mixed independently with 3.5 mL of soft MRS agar (0.75%), overlaid on Muller-Hilton agar plates, and incubated at 37°C for 2 h. Wells (diameter 6 mm) were punched in the agar layer aseptically, and the CE was placed in the wells, incubated at 37°C, and subsequently examined for inhibition zones at 48 h. All experiments were performed in triplicate. The results were expressed as mean ± SD. As a negative control, MRS broth has been used. For comparison, the CE of *L. plantarum* UTNGt2 has been used (Tenea and Ortega, 2021).

### The Ultrastructural and Morphological Changes Examination of *Salmonella enterica* subsp. *enterica* ATCC51741 Upon Peptide-Protein Extract Treatment

Cell culture at the exponential phase of *S. enterica* ATCC51741 (1 × 10^6^ CFU/mL) was treated with 1 × MIC (minimum inhibitory concentration) of PC for 6 h at 37°C following the procedure as previously described (Tenea et al., 2020). PC was obtained as described (Tenea et al., 2022). The MIC was defined as the minimum peptide concentration that inhibits 90% of the target cells after counting the viable bacteria in plate count after treatment with the PC at different concentrations (ranged from 400 to 9,600 AU/mL) compared with bacteria grew without PC added. The MIC (1X) for UTNGt21A was determined as 800 AU/mL. The samples preparation for TEM and SEM was performed following the procedure as described (Tenea et al., 2022). For TEM The grids (10 random sections per treatment) were examined using the Tecnai G2 F20 transmission electron microscope (FEI Company, Hillsboro, OR, United States). For SEM analysis, the samples were fixed on graphite tape and a thin coating of gold of approximately 24.5 nm was applied to each sample using a DENTON VACUUM Desk IV equipment (DENTON VACUUM, Austin, TX, United States) and subsequently analyzed in a high vacuum scanning electron microscope to obtain high-resolution images. The secondary electron detector was used to evaluate the morphology and topography of the samples. The samples were examined using JSM-6490 LV Scanning Electronic Microscopy equipment (JEOL, JSM, MA, United States).

## Results and Discussion

### General Genome Features

**FIGURE 1:**
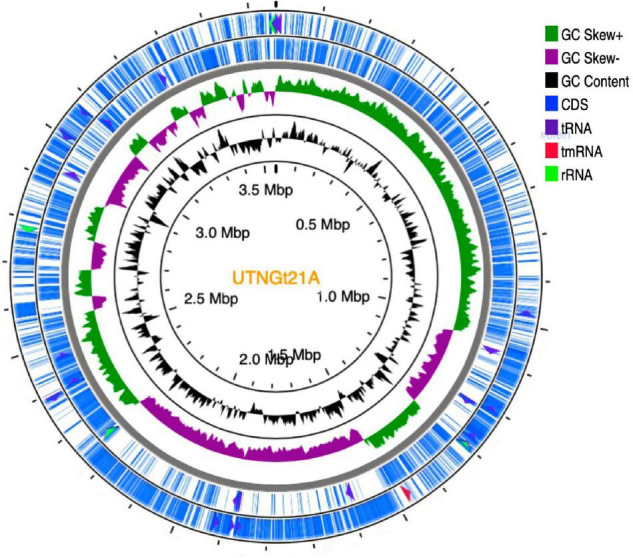
Circular genome diagram of *L. plantarum* UTNGt21A. From the inner circle to the outer ring: the first ring shows the location of the genome; the second depicts the GC skew (G + C/G – C); the third ring depicts the GC content; the fourth (forward strand) and fifth ring (reverse strand) showed the gene annotation with Prokka (the sites of CDSs/rRNA/tRNA/tnRNA on the genome are marked). CDS, coding sequence.

The genome sequencing and characterization of a novel *L. plantarum* strain UTNGt21A originated from *S. quitoense* wild fruits are described. The general genome features are depicted in Table 1. According to the available genome information from the NCBI database, the median total genome length of *L. plantarum* strains is 3.253 (Mb) with a median protein count of 2,927 and GC% of 44.5. The UTNGt21A genome was larger than the reference *L. plantarum* WCFS1, and *L. plantarum* ATCC14917 (GCA_000143745.1), a commercial probiotic strain isolated from pickled cabbage but very closed to the genome of *L. plantarum* UTNGt2, a strain isolated previously from wild white cocoa (copoaso) fruits (Tenea and Ortega, 2021). A circular map of the UTNGt21A strain is depicted in Figure 1. EggNOG analyses revealed that the most abundant gene category (11.9%) was predicted for general function, 8.98% encoded for proteins involved in carbohydrate transport and metabolism, 8.48% for transcription, while 25.83% of genes encoding hypothetical proteins were categorized to have an unknown function (Table 2). The genome annotation indicated that 3,209 proteins (3,168 proteins, Single EggNOG, and 41 Multi EggNOG) were matching EggNOG DB. No hit was found for 240 hypothetical proteins. Although these strains share a common genetic repertoire with proteins involved in replication, transcription, translation, energy production, carbohydrate transport, and metabolism, a disproportionate distribution of COG categories among *L. plantarum* strains has been observed (Botta et al., 2017). These results agree with previously published reports (Huang et al., 2020). In addition, one copy of the 23S rRNA gene of 2,919 nucleotides in length was detected in the genome of the native strains, while the reference WCFS1 showed five copies of 23S rRNA. Alignment with the NCBI database indicated that this fragment showed 100% identity with the 23S rRNA gene of *L. plantarum* DSM20174 isolated from pickled cabbage. The variable number of this gene might be inherent *via* horizontal change transfer in the environment they originate, thus indicating the plasticity of the *L. plantarum* genome (Huang et al., 2020).

**TABLE 1:** Genome features of *L. plantarum* UTNGt21A compared with the references *L. plantarum* WCFS1, *L. plantarum* ATCC14917, and *L. plantarum* UTNGt2 strains.

Strain	*L. plantarum* UTNGt21A	*L. plantarum* UTNGt2	*L. plantarum* ATCC14917	*L. plantarum* WCFS1
Source	*Solanum quitoense* (Lam.) wild fruit	Theobroma grandifolium (cocoa) wild fruit	Pickled cabbage	Human saliva
Genome length (bp)	3,558,611	3,540,752	3,198,761	3,308,274
Plasmids	None	None	None	3
GC content (%)	43.96	44.53	44.5	45.6
Total number of genes	3,524	3,115	3,040	3,174
Coding genes	3,471	3,052	2,942	3,063
tRNA number of assembled genome	68	57	67	70
rRNA number of assembled genome	6	5	16	15
tmRNA number of assembled genome	1	1	4	3
CRISPR-Cas array*	0	4	0	0
Prophage (intact region)**	3	1	2	4
Antibiotic acquired genes***	None	None	None	None
Pathogenicity****	Non-human pathogen	Non-human pathogen	Non-human pathogen	Non-human pathogen

*CRISPRFinder (https://crisprcas.i2bc.paris-saclay.fr/CrisprCasFinder/Index); **PHAge Search Tool Enhanced Release (PHASTER) (http://phaster.ca); ***ResFinder 4.1 (https://cge.cbs.dtu.dk/services/ResFinder/); ****PathogenFinder. (http://cge.cbs.dtu.dk/services/PathogenFinder/).

**TABLE 2:** EggNOG category distribution of functional annotation results.

EggNOG	Description	Count	Ratio (%)
J	Translation, ribosomal structure, and biogenesis	150	4.6140
A	RNA processing and modification	0	0.0000
K	Transcription	276	8.4897
L	Replication, recombination, and repair	173	5.3214
B	Chromatin structure and dynamics	0	0.0000
D	Cell cycle control, cell division, and chromosome partitioning	25	0.7690
Y	Nuclear structure	0	0.0000
V	Defense mechanisms	74	2.2762
T	Signal transduction mechanisms	78	2.3993
M	Cell wall/membrane/envelope biogenesis	181	5.5675
N	Cell motility	3	0.0923
Z	Cytoskeleton	0	0.0000
W	Extracellular structures	0	0.0000
U	Intracellular trafficking, secretion, and vesicular transport	25	0.7690
O	Posttranslational modification, protein turnover, and chaperones	65	1.9994
C	Energy production and conversion	107	3.2913
G	Carbohydrate transport and metabolism	292	8.9819
E	Amino acid transport and metabolism	205	6.3058
F	Nucleotide transport and metabolism	85	2.6146
H	Coenzyme transport and metabolism	65	1.9994
I	Lipid transport and metabolism	61	1.8763
P	Inorganic ion transport and metabolism	140	4.3064
Q	Secondary metabolites biosynthesis, transport, and catabolism	19	0.5844
R	General function prediction only	387	11.9040
S	Function unknown	840	25.8382
Total	–	3,251	100

*Count: number of genes; ratio (%): % of genes.*

### Taxonomic Classification and Phylogeny

**FIGURE 2:**
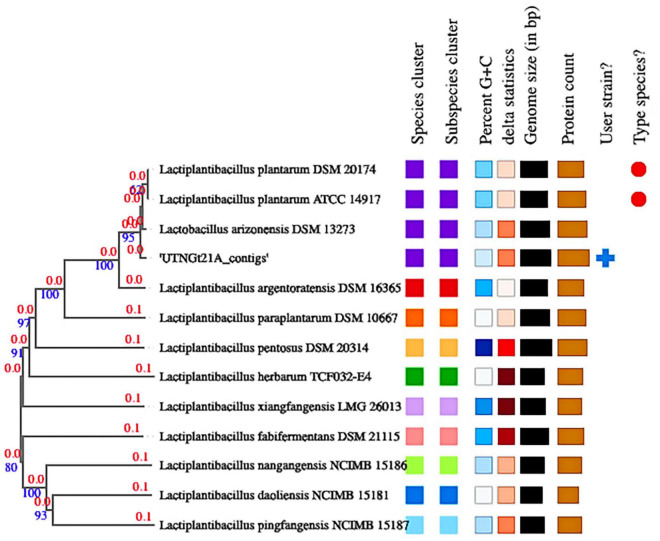
Phylogenetic tree based on TYGS result for the UTNGt21A whole-genome data set. Branch lengths are scaled in terms of GBDP (genome BLAST distance phylogeny method) distance; numbers above branches are GBDP pseudo-bootstrap support values from 100 replications. Leaf labels are annotated by affiliation to (1) species; (2) subspecies clusters; (3) genomic G + C content (min 43.5–46.31); (4) δ values (min 0.077–max 0.185); (5) overall genome sequence length (2.62–3.62 Mbp); and (6) number of proteins (2,418–3,471).

The proportion based on the genus level as the result of the best hit (BLASTN analysis) for the entire contig was 100% matching *Lactobacillus* (Supplementary Table 4). The ANI values were 99.27% nucleotide identity and 83.81% alignment coverage with the genome of *L. plantarum* UTNGt2 (Tenea and Ortega, 2021) and 99.00% nucleotide identity and 84.04% alignment coverage with the reference strain *L. plantarum* WCFS1, ranked according to the highest nucleotide identity (Supplementary Figure 1). Based on these analyses, the UTNGt21A isolate was assigned *L. plantarum*. Determination of the closest type of strain genomes was done by comparing the UTNGt21A genome against all type strain genomes available in the TYGS database *via* the MASH algorithm (Ondov et al., 2016), a fast approximation of intergenomic relatedness (Meier-Kolthoff et al., 2013). The strains with the smallest MASH distances were automatically selected. Consequently, the precise distance using the genome BLAST distance phylogeny approach (GBDP) under the algorithm “coverage” and distance formula was calculated (Farris, 1972). Based on DBDP, the 12 closest strain genomes were selected from the database. Type strains used in this dataset are shown in Supplementary Table 5. Branch support was inferred from 100 pseudo-bootstrap replicates each. The trees were rooted at the midpoint and visualized with PhyD3 (Kreft et al., 2017). The whole-genome alignment results placed the UTNGt21A strain in the same branch with *Lactobacillus arizonensis* DSM13273 and at a very close distance from *L. plantarum* ATCC14917 and *L. plantarum* DSM20174 (Figure 2). TYGS analysis results confirmed the identification results.

### Genome Stability

Neither CRISPR nor associated Cas sequences were annotated for the strain UTNGt21A with the CRISPRFinder web tool (Table 1). CRISPR/Cas system-associated genes were previously found in the UTNGt2 genome (Tenea and Ortega, 2021), but not the references *L. plantarum* WCFS1 and *L. plantarum* ATCC14917 strains. Besides, EggNOG analysis indicated that about 2.27% of proteins are involved in defense mechanisms. However, the elongation factor Tu (*tuf*) was annotated in the genome of UTNGt21A with 94.4% identity. This factor protects against bacteriophage invasion by blocking the multiplication of entry phages (Arellano et al., 2020). Although the CRISPR/Cas system is an adaptive defense mechanism detected in bacteria and might prevent the strains to gain resistance to antimicrobials and pathogenicity *via* horizontal transfer of genes (Rodrigo-Torres et al., 2019), the results indicated that UTNGt21A is a stable strain as no acquired mobile elements were detected. No plasmids were predicted in the genome of previously characterized lactic bacteria isolated from wild tropical fruits (Tenea et al., 2020). Most plasmids detected in lactic bacteria are cryptic, nonetheless, their presence or absence has no visible effect on growth, adaptation, and other properties (Turner et al., 2004). Their presence might constitute a potential safety risk due to the high prevalence of transferable antibiotic resistance to commensal and pathogenic bacteria, including those in the gastrointestinal tract (Obeng et al., 2016).

Prophage elements are commonly prevalent in bacteria, and they are related to species adaptation to various ecological niches (Gueimonde et al., 2013). A total of nine prophage regions were detected using the PHASTER web server, from which four were intact and five incomplete regions (Supplementary Figure 2). No questionable prophages were predicted. The largest intact prophage region detected within the contig 7 was PHAGE_Lactob_Sha1_ NC_019489 (10) of 47.8 Kb in length (Supplementary Table 6). This conserved prophage Sha1 was previously detected in the UTNGt2 strain but was absent in the reference WCFS1 genome. Previous studies suggested that these prophages might provide benefits during fermentation as they originated from plant material while the reference strain originates from human saliva (Rychen et al., 2018).

### Safety Characteristics

Safety evaluation is an essential step when selecting a new probiotic candidate (Leuschner et al., 2010). Microorganisms can acquire genes from other organisms sharing the same microenvironment, which can increase virulence, pathogenicity or boost antimicrobial drug resistance (Jia et al., 2017). The results from the CARD database indicated that UTNGt21A did not carry any strict or perfect antibiotic resistance genes. A total of 36 predicted genes were categorized by Drug Class, Resistance Mechanism, and AMR gene family (Supplementary Figure 3). No acquired antimicrobial-resistant genes were detected based on the RGI analysis. Although with CARD webserver one gene was predicted within an aminoglycoside drug class, and two genes were predicted within the glycopeptide class of antibiotics, this could not be confirmed by Prokka and EggNOG annotation neither by ResFinder analysis. Early studies reported that several strains from the *Lactobacillus* genus are vancomycin-resistant as they contain d-Ala-d-lactate in their peptidoglycan rather than the d-Ala–d-Ala dipeptide (Rychen et al., 2018). Two genes, *vanS*, and *vanR* encoding for a sensor histidine kinase VanS (40.1% identity) and a regulatory protein VanR (51.1% identity) were predicted with CARD but not EggNOG. The intrinsic vancomycin resistance was identified in a wide variety of bacterial species, including the Gram-positive *L. plantarum* strains (Deghorain et al., 2007). Analysis of the reference genome WCFS1 revealed the presence of a gene homologous to the resistance gene *van*X (lp_0769; designated *aad* for d-alanyl-d-alanine dipeptidase) (Kleerebezem et al., 2003). A recent report on *L. plantarum* strain Y44 indicated that the genome contains more than 30 intrinsic *van* genes (Gao et al., 2020). Besides, a gene *cat*A encoding for chloramphenicol resistance, but not erythromycin and bacitracin resistance genes were detected in the UTNGt21A genome. Likewise, complementary *in vitro* analysis indicated that the UTNGt21A strain was sensitive to all antibiotics tested (Supplementary Table 7). The resistance to chloramphenicol, erythromycin, and bacitracin was also reported in the reference WCFS1 strain as well as other *L. plantarum* strains. Two tetracycline resistance genes, *tet*A (tetracycline-resistant protein class B) and *tet*O (COG0408) were annotated in the UTNGt21A genome. These genes belong to the TetM/TetW/TetO/TetS family containing tetracycline resistance ribosomal protection proteins shared by several *Lactiplantibacillus* species including the reference WCFS1 strain (Kleerebezem et al., 2003). Nonetheless, lactobacilli innate resistance to antibiotics is not considered a safety concern, therefore according to the Rychen et al. (2018), strains intended to be used in probiotics for humans or additives for animal feed should not carry transferable antimicrobial resistance genes.

By employing the VFDB database with stringent criteria (>80% identity and >60% coverage), no virulence genes were predicted, while using less stringent criteria of >40% identity and >60% coverage, several genes were predicted as putative virulence factors (Supplementary Table 8). No genes encoding for virulence determinants (i.e., gelatinase, aggregation substance, and adhesion of collagen), were predicted within the UTNGt21A genome. Besides, a putative gene (*eno*) encoding for enolase with 50.1% identity and hemolysin III with 41.9% were predicted in the genome of UTNGt21A with VFDB. Three putative genes (*eno_1*, *eno_2*, and *eno_3*) encoding for enolase were annotated by EggNOG DB, while hemolysin failed to be detected by EggNOG DB annotation. Enolase is essential for the degradation of carbohydrates *via* glycolysis, by catalyzing the reversible conversion of 2-phosphoglycerate into phosphoenolpyruvate (Altermann et al., 2005). Among lactobacilli, enolase was found to bind to extracellular matrix proteins such as collagen, mucin, fibronectin, and laminin, therefore it was proposed that these commensal bacteria use surface-associated enolases as adhesins to colonize the host (Castaldo et al., 2009; Sanchez et al., 2009). Thus, is considered as a moonlighting protein that adheres to the gut epithelium, a desirable attribute of potential probiotic strains (Spurbeck et al., 2015). The presence of hemolysis genes was reported early in some probiotic strains such as *L. plantarum* BCC9546, 299V, JDM1 (Swain et al., 2014). Therefore, there is no proof of its expression to conclude its virulence. Nonetheless, when performed *in vitro* hemolysis analysis using growth media with 5% defibrinated sheep blood, no hemolysis was detected for the UTNGt21A strain (data not shown). The remaining gene matches were related to genes involved in the cellular processes, adaptation, and biogenesis of cell membrane. Thus, these genes might be useful to the strain as no pathogenicity was detected, therefore we concluded that UTNGt21A is a safe strain.

### Structural Biosynthetic Gene Clusters Organization

**FIGURE 3:**
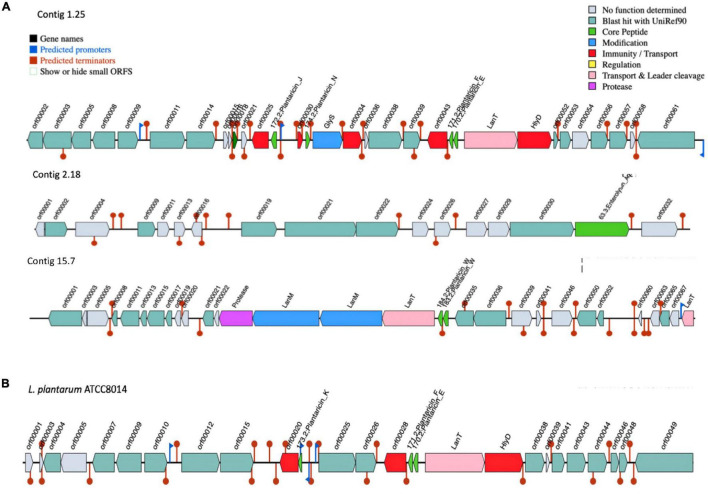
BGCs organization. **(A)** *L. plantarum* UTNGt21A. **(B)** *L. plantarum* ATCC8014. Genes with function determined from left to right (gene name, function, and locus tag): contig 1.25: orf00018: ComC, bacteriocin Class II with double-glycine leader peptide; orf00025, putative bacteriocin immunity protein; 172.2, plantaricin_J; orf00030, P71462_LACPL immunity protein PlnM; 174.2, plantaricin_N; GlyS: GlyS, glycotransferase family 2 protein (PlnO); 171.2, plantaricin_F; 170.2, plantaricin_E; LanT, bacteriocin ABC-transporter, ATP-binding and permease protein PlnG; HlyD, accessory factor for ABC-transporter PlnH; contig 2.18: orf00002, cystathionine beta-lyase PatB of *B. subtilis* (strain 168); orf00009, phosphatase YwpJ of *B. subtilis* (strain 168); orf00019, putative AgrB-like protein; orf00021, bacteriocin production related histidine kinase; orf00022, accessory gene regulator protein A of *Staphylococcus epidermidis* (strain ATCC 12228); orf00030, exo-glucosaminidase LytG of *B. subtilis* (strain 168); 63.3, enterolysin_A; contig 15.7: Protease, epidermin leader peptide-processing serine protease EpiP of *Staphylococcus epidermidis*; LanM (orf00025), lantibiotic modifying enzyme; LanM (orf00027), lantibiotic mersacidin modifying enzyme; LanT (orf00028), lantibiotic mersacidin transporter system; 184.2, plantaricin_W (alpha); 183.2; plantaricin_W (beta); orf00036, bacteriocin production related histidine kinase; orf00065, protein MesC of *Leuconostoc mesenteroides*; LanT (orf00039), ABC-type bacteriocin transporter; ATCC8014: 171.2, plantaricin_F; 170.2, plantaricin_E; LanT, bacteriocin ABC-transporter; HlyD, accessory factor for ABC-transporter PlnH; orf00010, bacteriocin IIc; 173.2, plantaricin_K. Red blocks, immunity and transport; green arrow, core peptide; pink block, transport and leader cleavage; blue block, peptide modifications; gray blocks, no function determined.

The gene clusters involved in bacteriocin production vary considerably in size, composition, and gene order across the genomes of *L. plantarum* strains (Goel et al., 2020). Early reports indicated that *L. plantarum* strains isolated from different environments are producing lantibiotic and/or non-lantibiotic bacteriocins (Rodrigo-Torres et al., 2019). Genome analysis by dedicated software tools, such as BAGEL4, identified three areas of interest (AOI) regions within the genome of UTNGt21A genome (Figure 3A). These BGCs were located within contig 1.2 (start at 314201, end at 341716) of the plantaricin_N class, contig 2.13 (start at 329768, end at 341294) of the enterolysin_A class, and contig 15.0 (start at 45593, end at 72493) of plantaricin_W alpha and plantaricin_W beta, a lanthipeptide Class II bacteriocin. The AOI of contig 1.2 resides with plantaricin_E (bit score = 112.464), plantaricin_F (bit score = 107.071), plantaricin_N (bit score = 108.227), and plantaricin_J (bit score = 100.138) belonging to Subclass IIB, a two-peptides, and Subclass IID of unclassified, bacteriocins. The reference WCFS1 harbors one AOI region with a *pln* cluster (Tenea and Ortega, 2021). Besides, the *pln* cluster of contig 1.2 showed almost the same organization as the *pln* cluster of the reference *L. plantarum* ATCC8014, a bacteriocinogenic strain (Figure 3B). In previous research, it has been shown that *L. plantarum* ATCC 8014 presents probiotic potential based on its inhibitory activity against *Clostridium* spp. (Monteiro et al., 2019). Although both UTNGt21A and ATCC8014 lack *pln*A gene encoding peptide involved in the transcription of the five operons of *pln* locus, the inhibitory activity was not lost as observed by the *in vitro* antimicrobial assays. Considering that both strains originated from raw fruit and plant material (naranjilla, corn silage), they may adapt their metabolism to stressful environmental conditions and interactions with other microbes comparting the same niches; however, the expression of the bacteriocins might be controlled by extracellular signals and their expression contribute to the overall inhibitory activity against the spoilage and poisoning of raw vegetables and fruits (Rizzello et al., 2014). Besides, in recent complementary genome analysis to identify antimicrobial gene cluster variants, 12 *pln*A downstream variants (located at 3′of gene) were annotated in the UTNGt21A genome. Besides, lacks the *pln*K gene (involved in the synthesis of bacteriocins), nonetheless, seven gene variants (four missense variants, two synonymous variants, and one initiator codon variant) were annotated in the genome of UTNGt21A (Tenea, 2022). The impact of these gene variants on gene expression and inhibitory activity cannot be predicted at this point, but we speculate that these variants might contribute to compensating or strengthening the overall antimicrobial activity. In a recent study, we showed that peptide-proteins extract from UTNGt21A applied as single or in combination with other antimicrobials induce the cell death of two multidrug-resistant pathogens (Tenea et al., 2022). Besides, UTNGt21A contains various ORFs similar to miscellaneous bacteriocins, such as ComC bacteriocin (orf00018), a Class II bacteriocin with double-glycine leader peptide; orf00025 encoding for a putative bacteriocin immunity protein; orf00034, encoding for a P71468_LACPL PlnI, a cognate immunity protein-containing CAAX-like protease; orf00038, a bacteriocin production-related histidine kinase; and three ORFs (orf00052, orf00056, and orf00057) encoding for a truncated plnS, a plantaricin detected in *L. plantarum* multispecies, and a CPBP family of intramembrane metalloproteases similar to CAAX prenyl protease 2, which proteolytically removes the C-terminal three residues of farnesylated and geranylated proteins. Enterolysin_A (contig 2.13) is a Class III bacteriocin, a heat-labile bacteriocin with a high inhibitory spectrum detected in *Enterococcus faecalis* (Nielsen et al., 2003). BLAST analysis of enterolysin_A protein sequences against the protein database indicates a specific hit to the M23 family of metallopeptidases (pfam01551) known as beta-lytic metallopeptidases identified in various *Lactobacillaceae.* These peptidases are Gly-Gly endopeptidases. Four residues that compose this conserved feature have been mapped to the query sequence. Based on BAGEL 4 analysis, neither ATCC8014, WCFS1 or UTNGt2 harbored enterolysin_A gene cluster. A putative enterolysin_A was predicted previously in the genome of the *Weissella cibaria* UTNGt21O strain (Tenea et al., 2020), sharing the same origin as the UTNGt21A strain. Based on these results, we speculated that this bacteriocin could be acquired during a horizontal transfer gene between species habituating the same microenvironment. Within contig 15.0, two copies of the LanM and one copy of protease encoding genes were detected upstream of LanT (lantibiotic mersacidin transport system). Besides, plantaricin_W (alpha and beta), a two-peptide lantibiotic, was annotated in the UTNGt21A genome but not WCFS1, ATCC8014, and UTNGt2 genomes (Tenea and Ortega, 2021). Previous research on detection of genes responsible for bacteriocin synthesis in several *L. plantarum* strains showed that none of the strains harbored *pln*W genes, considered as a relatively rare bacteriocin among lactobacilli which inhibits many Gram-positive bacteria (Holo et al., 2001). LanM is a lantibiotic modifying enzyme, harboring a domain DUF4135 (of approximately 380 amino acids in length) of unknown function. This domain may be involved in the synthesis of a lantibiotic compound. LanM of the query UTNGt21A sequence contains three zinc-binding sites with the conserved domain LanM-like. BLASTP analysis indicated that the protease is a peptidase_S8_lantibiotic specific protease domain-containing protein, similar in structure to epidermin leader peptide-processing serine protease EpiP from *Staphylococcus epidermidis* (Siezen and Leunissen, 1997). In addition, EggNOG analysis confirmed the presence of a serine protease gene, *nis*P, a nisin leader peptide-processing serine protease with 28% sequence identity to nisin cyclase from *Lactococcus lactis* in the genome of UTNGt21A. Three copies of the LanT gene encoded for the accessory factor of ABC-transporter were found within contig 1.25 (one copy) and contig 15.7 (two copies) of UTNGt21A. LanT impel as a maturation protease and as an exporter for quorum-sensing like *E. coli* colicin V secretion/processing ATP-binding protein CvaB, or *Bacillus subtilis* SPBc2 prophage-derived sublancin-168-processing and transport ATP-binding protein SunT. The ABC bacteriocin/lantibiotic exporters contain an N-terminal double-glycine peptidase domain and are involved in defense mechanisms (Havarstein et al., 1995). Furthermore, several species-specific lactococcin-G-processing encoding genes (*lagD_1, lagD_2, lagD_3, lagD_4*, and *lagD_5*) and transport ATP-binding proteins (*lag*D) were annotated with EggNOG (Supplementary Table 9). These ABC transporters were early identified in *L. lactis* species and belong to Class IIb of bacteriocins (Havarstein et al., 1995; Wu et al., 2021). BLAST against the core peptide database hit enterocin_W_alpha of *E. faecalis* NKR-4-1 (bit score = 30.4116), patellamide_C_patE1 and patellamide_A_patE1 a subclass of cyanobactin from *Prochloron* sp. (bit score = 28.8758), ulithiacyclamide_patE2 (bit score = 28.4906) from *Prochloron* sp., patellamide_C_patE2 (bit score = 28.4906), ubericin A (bit score = 33.113) from *Streptococcus uberis*, and bacteriocin_LS2chaina (bit score = 28.1054) from *L*actobacillus *salivarius* BGH01. Overall, these features suggested that the UTNGt21A strain is an antimicrobial producer strain showing a complex and unique BGCs organization divergent from the reference WCFS1, ATCC8014, and UTNGt2 strains (Tenea and Ortega, 2021). We suggest that the general inhibitory capacity against a specific target species may be ligated with the efficacy of the peptide-protein mixture released by the LAB strain and interconnected with the gene organization and their specific mode of action. Further comparative genome analysis to identify bacteriocin gene variants (single-nucleotide polymorphisms) of native strains is under investigation and might help to understand their overall inhibitory strength (Tenea, 2022).

### Prediction of Primary Metabolites

**FIGURE 4:**
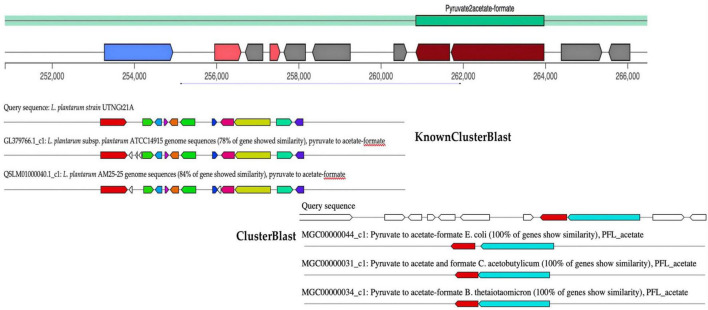
gutSMASH run for query sequence (UTNGt21A) predicting one MGC region, type pyruvate to acetate-formate. The KnownClusterBlast results showed the gene similarity from this branch with other genes of *Lactobacillus* species from database. The ClusterBlast output shows that this gene cluster does not have homologous MGCs among another *Lactobacillus* present in the database. Genes marked with the same color are interrelated. White genes have no relationship.

Microorganisms are producing a wide range of natural substances such as primary and secondary metabolites in the ecosystem they are habituating. gutSMASH tool was recently proposed to evaluate anaerobic bacterial genomes for primary specialized MGCs (Pascal Andreu et al., 2021). The primary metabolites (amino acids, nucleotides, and fermentation end products such as ethanol and organic acids) allow bacteria to either colonize specific micro-niches such as the gut or interact with other microbes modulating the host metabolism, immunity, and homeostasis, thus they are of great interest. Although some of these molecules are produced by low-abundance bacteria, they can reach high concentrations in the gut as well as in blood plasma (Funabashi et al., 2020). Among them, trimethylamine derived from carnitine and choline metabolism was found to be associated with an increased risk of cardiovascular disease (Rath et al., 2020). In addition, short-chain fatty acids (SCFAs) have been found to positively impact human health, their most abundant representatives are acetate, butyrate, and propionate (Wong et al., 2006). From the query genome sequence of UTNGt21A, only one MGC region, pyruvate to acetate-formate type (region 3.1), with a total of 15,660 nucleotides (location: 250,855–266,484 nucleotides) was detected (Figure 4). This region belongs to SCFAs, and their most abundant representatives are acetate, butyrate, and propionate (Donia and Fischbach, 2015). The KnownClusterBlast region output indicated that this region showed 78% similarity with *L. plantarum* ATCC14917 and 84% with *L. plantarum* AM25 strain. The ClusterBlast output showed that there is no homolog MGCs to *Lactobacillus* species in the database. Genes from this region have 100% similarity with pyruvate to acetate-formate of *E. coli*, *Clostridium acetobutylicum*, and *Bacteroides thetaiotaomicron*. Pyruvate formate lyase and acetate kinase are essential for anaerobic growth (Hasona et al., 2004). Besides, two genes, *pflA* and *pflB*, encoding for pyruvate formate lyase (COG1180, 72% identity) and formate acetyltransferase (COG1882, 82% identity) were annotated with the EggNOG. In addition, pyruvate is a key substance in the central carbon metabolism of lactobacilli and its content is essential for the synthesis of organic acids (Wang et al., 2021). The production of organic acids such as acetate, citrate, lactate, and formate by LAB strains and their importance in the final flavor of some products was earlier investigated (Nuryana et al., 2019).

### Production of Secondary Metabolites

**FIGURE 5:**
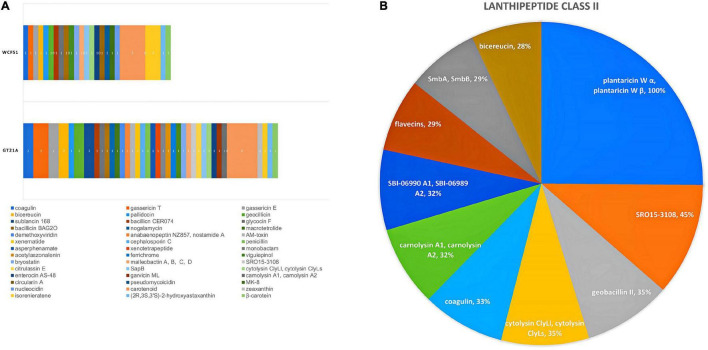
**(A)** Comparison between the secondary metabolite clusters detected in the UTNGt21A genome and the reference *L. plantarum* WCFS1 strain. Different colors and numbers marked the compounds produced by each strain. **(B)** Pie chart depicting different compounds of lanthipeptide Class II predicted for UTNGt21A. The current region sequence similarity (%) with the reference MIBiG database is shown.

Secondary metabolites and RiPP-like molecules carry out very diverse functions as they actuate as antibiotics, antitumor agents, antifungals, antivirals, etc. (Chen et al., 2019). In the present study, the genome contigs were used as input in the antiSMASH web tool to predict the type and location of secondary metabolites produced by the UTNGt21A strain (Table 3). Eight regions were detected with a total of 44 RiPP-like, 10 lanthipeptide-class-II (RiPP-like), 13 terpenes, 9 NRP, 6 PKs, 3 not identified, and 1 alkaloid. A summary of predicted compounds, category, location, and similarity scores are shown in Supplementary Table 10. Among the RiPP-like category, some compounds such as coagulin, glycocin F were common between the UTNGt21A and the reference WCFS1 or UTNGt2 strains. Nonetheless, sublancin 168 was identified in the UTNGt21A genome only. Figure 5A depicts a comparison between the predicted compounds. Several lanthipeptide of Class II were detected in the UTNGt21A genome but not in the reference WCFS1 or UTNGt2 genomes (Figure 5B). The ClusterBlast output showed lower gene similarity (28%) with *L. plantarum* TMW 1.308 (NZ_CP021929). We speculate that the low similarity might be linked to their origin as this strain was isolated from fermented sausages (Nuryana et al., 2019). Among the lanthipeptides of Class II, the most similar gene cluster was plantaricin W (alpha and beta), showing 100% identity with plnC from several *L. plantarum* strains (Ehrmann et al., 2000). In addition, a bacteriocin showing 35% sequence similarity to geobacillin II from *Geobacillus thermodenitrificans* strain NG80-2, a thermophilic bacterium with a high inhibitory capacity, was predicted within the genome of UTNGt21A (Garg et al., 2012). Besides, a putative bacteriocin having 28% sequence similarity to bicereucin from *Bacillus cereus* SJ1 strain was predicted with antiSMASH. Agar diffusion growth inhibition assays demonstrated that this bacteriocin inhibits several Gram-positive bacteria (Cuadrat et al., 2018). These bacteriocins were not predicted in the reference WCFS1 and UTNGt2 genomes, suggesting that these molecules could be inherent to the ecological microenvironment origin, further analysis is required to prove this statement.

**TABLE 3:** Identified secondary metabolite biosynthetic gene clusters with antiSMASH using strictness “strict”*.

Contig. region	Type	Location (length)	ClusterBlast**/KnownClusterBlast gene similarity (%)
1.1	RiPP-like	326,984–339,134 nt. (total: 12,150 nt.)	92/not match
2.1	Cyclic lactone autoinducer	323,444–341,294 nt. (total: 17,850 nt.)	9/not match
3.1	Cyclic lactone autoinducer	9,652-30,357 (total: 20,705 nt.)	8/not match
3.2	RiPP-like	197,414–209,024 nt. (total: 11,611 nt.)	100/not match
4.1	NRPS	175,160–234,843 nt. (total: 59,684 nt.)	100/not match
9.1	T3PKS	149,146–178,013 nt. (total: 28,868 nt.)	74/not match
15.1	Lanthipeptide Class II	44,600–76,188 nt. (total: 31,589 nt.)	28/100 (plantaricin W α/β)
22.1	Terpene	8,390–29,271 nt. (total: 20,882 nt.)	69/not match

*Detects well-defined clusters containing all required parts; **% similarity with several Lactiplantibacillus plantarum strains from the database.

Usually, terpenes are produced by fungi and plant genomes but were reported in bacteria (Cuadrat et al., 2018). Zeaxanthin, one of the most frequent carotenoids found in nature (National Center for Biotechnology Information, 2021), was predicted in the UTNGt21A genome but not the reference WCFS1 and UTNGt2 genomes (Tenea and Ortega, 2021). The ClusterBlast output showed 50% similarity with the reference WCFS1 and 69% with *L. plantarum* strain TCI507 (NZ_CP054259). No similar known gene clusters were detected in the database. Among the predicted NRPS/PKS (polyketide synthase) compounds, anabaenopeptin NZ857, nostamide A, xenematide, asperphenamate, cephalosporin C, penicillin, xenotetrapeptide, monobactam, acetylaszonalenin, and AM-toxin were predicted in the UTNGt21A genome (Supplementary Table 10) but not in the reference WCFS1 or UTNGt2 strains. The ClusterBlast output showed 84% similar gene clusters with the reference WCFS1 and 90% with *L. plantarum* strain D1501 (NZ_CP028326). As well as terpenes, no similar known gene clusters were detected in the database. Cephalosporin C query sequence was similar with the cephalosporin C BGC (similarity score 0.30) from *Acremonium chrysogenum* ATCC11550, while penicillin was similar with penicillin BGC (similarity score 0.29) from *Penicillium chrysogenum*, as detected by the MIBiG comparison.

Non-ribosomal peptide synthases are multifunctional enzymes that synthesize many therapeutical peptides in bacteria and fungi *via* a template-directed, nucleic acid independent non-ribosomal mechanism with a distinct modular structure (Rodrigo-Torres et al., 2019). In this study, two NRPS/PKS modular domains were predicted for the UTNGt21A strain. Ctg4_200 domain is comprised of one AMP-binding (adenylation domain), and one thioesterase domain, while the ctg4_203 domain consists of five AMP-binding domains, one epimerization domain, and five condensation domains linking an L-amino acid to a peptide ending with an L-amino acid (condensation_LCL). BLASTN analysis of both domains showed 100% similarity with AMP-binding and amino-acid adenylation domain-containing peptides from *Lactobacillaceae* multispecies. These biomolecules might act as competitors in the ecological niches they are produced enhancing antimicrobial resistance by competing for space and nutrients, but their *ex vitro* behavior should be evaluated. In addition, within contigs 2 and 3, a cyclic lactone autoinducer region was predicted within the UTNGt21A genome but not in the reference WCFS1 and UTNGt2 genomes. The ClusterBlast of region 2.1 showed very low gene similarity (9%) with thiopeptides from *Bacillus* sp., while region 3.1 showed 8% similarity with lanthipeptides from *Enterococcus moraviens.* No similar known gene clusters were detected. In prokaryotic organisms, cyclic peptides are key components of signal transduction pathways such as quorum sensing. These molecules are small peptides or chemical signals, usually autoinducers, used by Gram-positive bacteria to measure the population density (Mull et al., 2018).

### RiPPMiner Genome Analysis Revealed the Presence of Diverse RiPPs Molecules

Unlike the antiSMASH computational algorithm, where the information is limited and mostly dependent on pre-annotated bacteriocin databases, RiPPMiner uses a machine learning model trained to retrieve RiPPs from sequenced genomes and then predict their class, structure, crosslinks, and cleavage sites for selected RiPP families, such as lanthipeptide, cyanobactin, lasso peptide and thiopeptide (Russell and Truman, 2020). In the present study, out of 1998 initially identified cluster hits, 44 clusters were classified as biosynthetic RiPP peptides after the removal of the overlap sequences. However, the genome of UTNGt21A contains 19 glycosins, 11 linaridin, 7 lanthipeptide B, and 7 lanthipeptide C_D (Supplementary Table 11). Although the number of detected RiPP molecules extracted with antiSMASH and RiPP miner was similar, the NRPS/PKS molecules were detected with antiSMASH only. Among RiPP molecules, glycocins were mainly found in *Firmicutes* and are the only ribosomally synthesized glycosylated bacteriocins discovered and characterized (Singh and Rao, 2020). These peptides display antimicrobial activity against methicillin-resistant *S. aureus* and food spoilage bacteria *L. monocytogenes*. In addition, glycocins exhibit immunostimulatory properties and make a promising source of new antibiotics and food preservatives analogs to nisin. From the input genome of UTNGt21A, diverse precursor peptides were predicted with RiPPMiner, but not their chemical structure. The glycocins showed different ORFs with several biosynthetic domains such as ABC transporter, alpha-beta hydrolase, flavoproteins, S-glycosyltransferase, and methyltransferases (Supplementary Table 11). Linaridins (linear arid peptides) represent a moderately sized RiPP class characterized by the presence of dehydrobutyrine (Dhb) on linear ribosomal peptides (Ma and Zhang, 2020). Being a relatively underexplored class of RiPP, the Dhb-installing enzyme function remains unknown. As well as glycocins, no structural organization could be predicted by the RiPPMiner web tool and the ORFs showed several biosynthetic domains such as ABC transporter, alpha-beta hydrolase, and methyltransferases. Moreover, the chemical structure, cleavage sites, and crosslinks were predicted for both lanthipeptide B and lanthipeptide C_D classes (Supplementary Table 11). Lanthipeptides are biosynthesized by LanM (InterPro family: IPR017146), a bifunctional lanthipeptide synthetase (Montalbán-López et al., 2021). The ORFs number diverged between the lanthipeptides and showed variable biosynthetic domains such as LanM_dehydratase, ABC transporters, kinase domains, and alpha-beta hydrolase. Taken together, the application of a targeted genome mining tool uncovered a diverse arsenal of RiPP molecules within the genome of the UTNGt21A strain. The interest in applying lanthipeptides as antimicrobial agents is rising, nonetheless, there is a strong need to enhance their production as their isolation from native sources is an expensive time-consuming process. Coupling the *in silico* prediction strategies with *in vitro* characterization will increase our understanding of how these compounds are synthesized in nature. Further genetic manipulation of the BGCs or a specific metabolic pathway might improve the production of different RiPP molecules from probiotic bacteria.

### Wide-Range of Inhibitory Activity Against Several Foodborne Pathogens

By a well-diffusion agar assay, the CE obtained from UTNGt21A and previously characterized *L. plantarum* UTNGt2 strain were screened for their antibacterial activity against nine different common pathogens including new isolates from local tropical juices sold in the market. The results indicated that CE obtained from both strains showed high inhibitory activity against all targets tested with the highest diameter of inhibition zone registered against *S. enterica* subsp. *enterica* ATCC51741 (20.33 ± 0.58 mm) followed by *E. hormaechei* UTNB3Sh1 (18 ± 00 mm) and *K. cowanii* UTNB2Sh1 (14 ± 00 mm) (Table 4). Likewise, CE from UTNGt2 inhibited *Enterobacter* and *Listeria*. Likewise, the CE from both strains inhibit *Shigella, Kosakonia*, and *Staphylococcus* strains. Although both strains exerted an almost similar spectrum of inhibition, the small difference might be related to the nature of the substances released in the extract and the target pathogen. Early studies demonstrated the inhibitory activity of bacteriocin-like substances from different *L. plantarum* is species-specific (Rocchetti et al., 2021). Our results were in concordance with our previous results indicating that both the spectrum and the strength of antibacterial property of LAB are strain-specific. Of particular interest, the strain UTNGt21A showed the strongest inhibitory activity against two isolated identified in commercial juices, *K. cowanii* UTNB2Sh1 and *E. hormaechei* UTNB3Sh1, however, we shall further test the inhibitory activity of the peptide-proteins combinations *ex vitro* against single or specific targets mixed in tropical juice matrices.

**TABLE 4:** Inhibitory spectrum of CE obtained from UTNGt21A and UTNGt2 strains.

Indicator strain	Diameter of the inhibition zone (mm)	
	UTNGt21A	UTNGt2
*Staphylococcus aureus* ATCC1026	9.17 ± 0.29	9.17 ± 0.29
*Staphylococcus aureus* ATCC43300	12.22 ± 0.58	9.17 ± 0.29
*Listeria monocytogenes* ATCC19115	12.67 ± 0.58	14.33 ± 0.58
*Shigella sonnei* ATCC25931	12.67 ± 0.58	14.67 ± 0.58
*Enterobacter hormaechei* UTNB3Sh1	18.33 ± 0.58	15.67 ± 0.58
*Kosakonia cowanii* UTNB2Sh1	14.67 ± 0.58	14.67 ± 0.58
*Shigella diphtheriae* UTNFa37-1	12.33 ± 0.58	12.33 ± 0.58
*Salmonella enterica* subsp. *enterica* ATCC51741	20.33 ± 0.58	14.33 ± 0.58
*Escherichia coli* ATCC25922	12.33 ± 0.58	14.33 ± 0.58
MRS broth (negative control)	(−)	(−)

*The mean (± SD) of the diameter of the inhibition zone (mm) is shown. (−) No inhibition*

### Protein Extract Induces Morphological and Ultrastructural Changes of *Salmonella enterica* subsp. *enterica* ATCC51741 Cells

**FIGURE 6:**
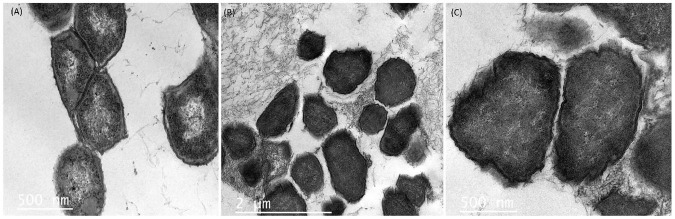
Transmission electronic microscopy images of *S. enterica* subsp. *enterica* ATCC51741. **(A)** Untreated bacteria. **(B,C)** Bacteria treated with PC from UTNGt21A at 1 × MIC for 6 h. Scale bars correspond to 500 nm and 1 μm.

**FIGURE 7:**
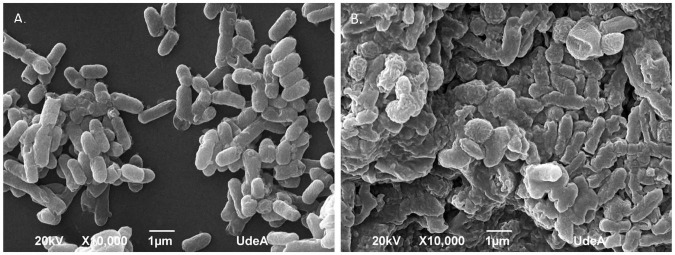
Scanning electronic microscopy of *Salmonella enterica* subsp. *enterica* ATCC51741. **(A)** Untreated bacteria; **(B)** cells treated with PC from UTNGt21A at 1 × MIC for 6 h. Scale bars correspond to 500 nm and 1 μm.

Efforts to reduce the drug-resistant microorganisms’ growth in foods using antimicrobial substances produced by LAB are provocative and complex undertakings. The morphological and ultrastructural cell modifications because of direct interaction between cells and the antimicrobial agent might depend on the identity of the agent tested as well as the concentration applied and time of exposure (Cushnie et al., 2016). From the agar-well experiment, we observed that the UTNGt21A extract showed the highest inhibitory activity against *S. enterica* ATCC51741, therefore we investigated the effect of PC on cell morphological and ultrastructural changes by both TEM and SEM analysis. Untreated cells showed normal morphology with intact cell walls (Figure 6A) and smooth surface and rod shape (Figure 7A). The cell treated with peptides (1 × MIC) displayed several irregular cells with undulated cell walls (Figures 6B,C). SEM showed deformed cells with wrinkles (Figure 7B). In a previous study, when treated *S. enterica* ATCC51741 with peptides originated from two *L. plantarum* strains, at least four simultaneous secondary events such as spheroplasts, DNA relaxation, vacuolation, and cells deformation were detected (Tenea, 2020). More recently, we showed that PCs treatment containing a combination between peptide-protein extracts from UTNGt21A and UTNGt28, a *Lactococcus* strain, induced several ultrastructural modifications of two multidrug-resistant pathogens (Tenea et al., 2022). In the current study, the results agreed with these studies confirming that the inhibitory effect might depend on the type and composition of the antimicrobial extract and the target. Considering these promising results, we shall further evaluate the inhibitory effect *ex vitro* in different foods.

## Conclusion

This study reports the whole genome characterization of *L. plantarum* strain UTNGt21A isolated from wild naranjilla fruits. Genome characterization and *in silico* approach-based analysis indicated that UTNGt21A is a stable and safe strain. Besides, the strain harbors genes encoding for various secondary metabolites, BGCs, RiPPs-molecules, PKs, NRPSs, and terpenes, versatile and diverse natural substances obtained from microbes of fruits ecosystems, which might confer a wide range of biotechnological benefits. The great diversity of the predicted RiPPs molecules detected throughout the genome suggests that lactic bacteria isolated from wild tropical fruits carry a rich repertoire of diverse molecules that could be inherent to their ecological microenvironment origin. Besides, the probiotic features, the antagonistic capacity against several food pathogens indicated that the antimicrobials produced by the UTNGt21A strain are promising candidates to be tested *ex vitro* as biocontrol agents against potentially harmful microorganisms during food processing and storage for the increasing of the shelf-life and safety of food products. Nonetheless, decoding the antimicrobial capability by using *in silico* analysis and further coupling with *in vitro* and *in vivo* characterization is required to prospect their use as an alternative to conventional antibiotics for a healthy gut, as well as to explore their technological properties as probiotics, or as novel therapeutics. Besides, in developing countries where the culture of consuming probiotics is very poor, the incorporation of *L. plantarum* strains into traditional non-dairy-based fermented foods or beverages might be a solution to resolve worsening health conditions. Nonetheless, there are many challenges ahead and the appropriate selection and characterization of probiotic microorganisms and the food matrices intended to be proven is essential.

## Data Availability Statement

The datasets presented in this study can be found in online repositories. The names of the repository/repositories and accession number(s) can be found in the article/Supplementary Material.

## Author Contributions

GT and PA contributed to the formal investigation and analysis. GT contributed to conceptualization, methodology, data curation, supervision, project administration, funding acquisition, writing—original draft preparation, and writing—review and editing. Both authors contributed to the article and approved the submitted version.

## Conflict of Interest

The authors declare that the research was conducted in the absence of any commercial or financial relationships that could be construed as a potential conflict of interest.

## Publisher’s Note

All claims expressed in this article are solely those of the authors and do not necessarily represent those of their affiliated organizations, or those of the publisher, the editors and the reviewers. Any product that may be evaluated in this article, or claim that may be made by its manufacturer, is not guaranteed or endorsed by the publisher.

## Footnotes

1
http://www.bioinformatics.babraham.ac.uk/projects/fastqc
