# Supplementary material for: Bioprospecting of Ribosomally Synthesized and Post-translationally Modified Peptides Through Genome Characterization of a Novel Probiotic Lactiplantibacillus plantarum UTNGt21A Strain: A Promising Natural Antimicrobials Factory
Source: Front Microbiol. 2022 Apr 6;13:868025. doi: 10.3389/fmicb.2022.868025 (PMC9020862; doi:10.3389/fmicb.2022.868025)
Supplement: Supplementary file 1 [file Data_Sheet_1.zip › Figure 1.DOCX]

**Supplementary Figure 1**. **A).** Percent identity heatmap resulted from ANI analysis. The cells corresponding to an ANI value of 95% and higher are stained red, indicating that the corresponding strains belong to the same species. The dendrograms (in green; above and on the left side), which were constructed by the simple linkage of the ANIm (ANI with MUMmer) percentage identities, correspond to the results of the clustering of the ANI values between the used strains (Pritchard et al., 2016). **B).** Alignment coverage heatmap. The isolates and species assignments as indicated at source are given as row and column labels. Cells in the heatmap corresponding to 75% coverage or greater are colored red. Color intensity fades as the comparisons approach 50% coverage. The dendrograms (in green; above and on the left side) of the heatmap correspond to strains assignments for each isolate in the analysis. ANI: Average Nucleotide Identity

A)

UTNGt2

UTNGt21A

UTNGt2

UTNGt21A

**B)**

UTNGt2

UTNGt21A

UTNGt2

UTNGt21A

UTNGt2

UTNGt21A

UTNGt2

UTNGt21A
