# Supplementary material for: Bioprospecting of Ribosomally Synthesized and Post-translationally Modified Peptides Through Genome Characterization of a Novel Probiotic Lactiplantibacillus plantarum UTNGt21A Strain: A Promising Natural Antimicrobials Factory
Source: Front Microbiol. 2022 Apr 6;13:868025. doi: 10.3389/fmicb.2022.868025 (PMC9020862; doi:10.3389/fmicb.2022.868025)

**Supplementary Figure 2.** Prophage region mapping predicted in the genome of UTNGt21A strain. Legend: green: intact region; red: incomplete.


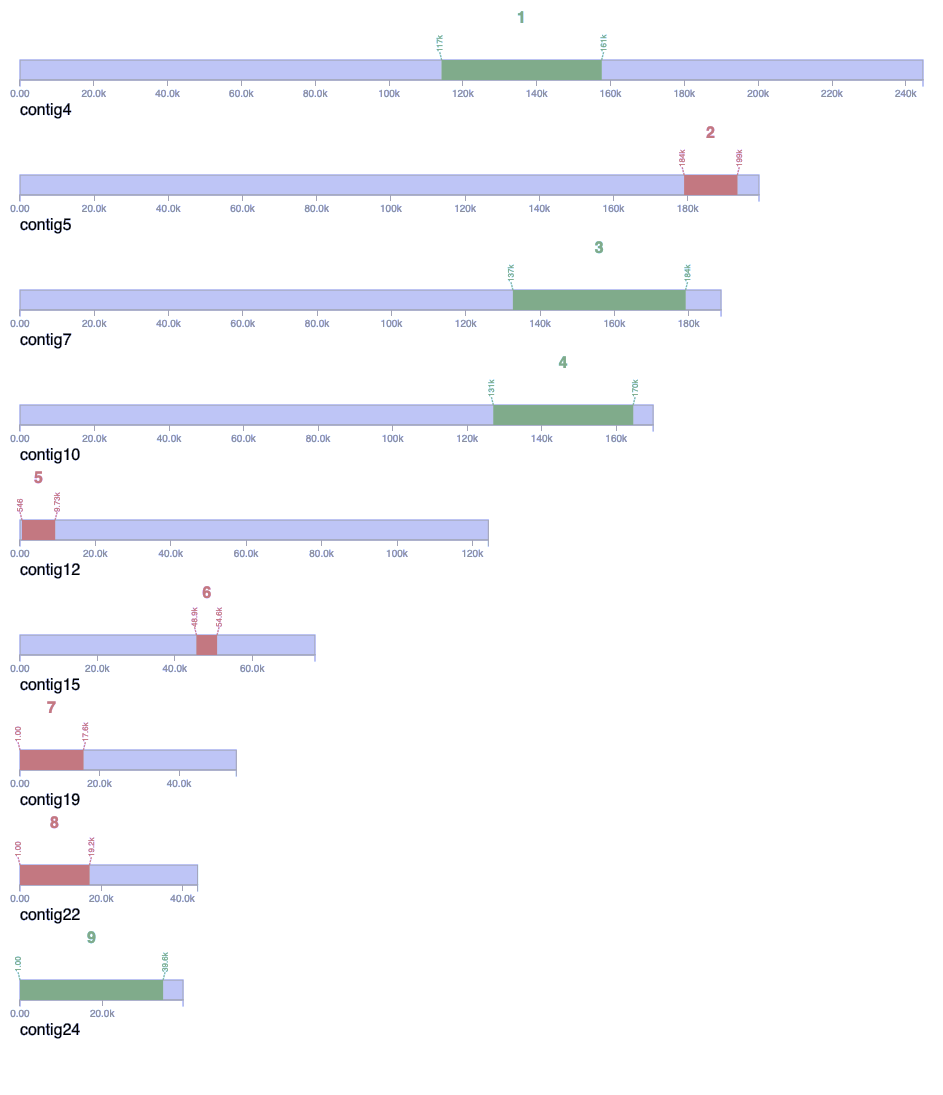

Supplement: Supplementary file 1 [file Data_Sheet_1.zip › Figure 2.DOCX]
