# Supplementary material for: Bioprospecting of Ribosomally Synthesized and Post-translationally Modified Peptides Through Genome Characterization of a Novel Probiotic Lactiplantibacillus plantarum UTNGt21A Strain: A Promising Natural Antimicrobials Factory
Source: Front Microbiol. 2022 Apr 6;13:868025. doi: 10.3389/fmicb.2022.868025 (PMC9020862; doi:10.3389/fmicb.2022.868025)
Supplement: Supplementary file 1 [file Data_Sheet_1.zip › Table 1.DOCX]

**Supplementary Table 1.** Assembly summary results

| **Sample** | **Contigs** | **Total contig bases** | **N50** | **Max length** | **Min length** | **Mean length** |
| --- | --- | --- | --- | --- | --- | --- |
| UTNGt21A | 38 | 3,558,611 | 188,693 | 447,978 | 1230 | 93,647 |

Contigs: The number of contigs assembled

Total bases of contigs: The total length of contigs

N50: 50% of all contig bases come from contigs longer than this value

Max length: The length of the longest contig

Min length: The length of the shortest contig

Mean length: The average length of contigs assembled
