# Supplementary material for: Bioprospecting of Ribosomally Synthesized and Post-translationally Modified Peptides Through Genome Characterization of a Novel Probiotic Lactiplantibacillus plantarum UTNGt21A Strain: A Promising Natural Antimicrobials Factory
Source: Front Microbiol. 2022 Apr 6;13:868025. doi: 10.3389/fmicb.2022.868025 (PMC9020862; doi:10.3389/fmicb.2022.868025)
Supplement: Supplementary file 1 [file Data_Sheet_1.zip › Table 10.DOCX]

**Supplementary Table 10.** Summary of the biosynthetic gene clusters, type, and metabolic compounds detected with antiSMASH

| **Sample contig** | **Reference**  **MiBIG cluster** | **RiPP-Type** | **Sequence similarity (%)** | **Product** | **Compound(s)** | **Organism** |
| --- | --- | --- | --- | --- | --- | --- |
| contig 1.1 | [BGC0000617.1](https://mibig.secondarymetabolites.org/repository/BGC0000617/index.html#r1c1) | **RiPP-like** | 22.0 | RiPP | coagulin | *Bacillus coagulans* |
|  | [BGC0000619.1](https://mibig.secondarymetabolites.org/repository/BGC0000619/index.html#r1c1) |  | 17.0 | RiPP | gassericin T | *Lactobacillus gasseri* |
|  | [BGC0001602.1](https://mibig.secondarymetabolites.org/repository/BGC0001602/index.html#r1c1) |  | 17.0 | RiPP | gassericin-T | *Lactobacillus gasseri* |
|  | [BGC0001388.1](https://mibig.secondarymetabolites.org/repository/BGC0001388/index.html#r1c1) |  | 17.0 | RiPP | gassericin E | *Lactobacillus gasseri* |
|  | [BGC0001407.1](https://mibig.secondarymetabolites.org/repository/BGC0001407/index.html#r1c1) |  | 16.0 | RiPP | bicereucin | *Bacillus cereus SJ1* |
|  | [BGC0001931](https://mibig.secondarymetabolites.org/repository/BGC0001930/index.html#r1c1) |  | 16.0 | RiPP | pallidocin | *Aeribacillus pallidus* |
|  | [BGC0001862.1](https://mibig.secondarymetabolites.org/repository/BGC0001862/index.html#r1c1) |  | 16.0 | RiPP | geocillicin | *Aeribacillus pallidus* |
|  | [BGC0000558.1](https://mibig.secondarymetabolites.org/repository/BGC0000558/index.html#r1c1) |  | 16.o | RiPP | sublancin 168 | *Bacillus subtilis subsp. subtilis str. 168* |
|  | [BGC0001863.1](https://mibig.secondarymetabolites.org/repository/BGC0001863/index.html#r1c1) |  | 16.0 | RiPP, Terpene | bacillicn CER074 | *Bacillus mycoides* |
|  | [BGC0001861.1](https://mibig.secondarymetabolites.org/repository/BGC0001861/index.html#r1c1) |  | 15.0 | RiPP | bacillicin BAG2O | *Bacillus cereus BAG2O-1* |
| contig 2.1 | [BGC0001291.1](https://mibig.secondarymetabolites.org/repository/BGC0001291/index.html#r1c1) | **Cyclic lactone autoinducer** | 16.0 | RiPP | enterocin NKR-5-3B | *Enterococcus faecium* |
|  | [BGC0001229.1](https://mibig.secondarymetabolites.org/repository/BGC0001229/index.html#r1c1) |  | 16.0 | RiPP | SBI-06990 A1, SBI-06989 A2 | *Streptomyces bingchenggensis BCW-1* |
|  | [BGC0000575.1](https://mibig.secondarymetabolites.org/repository/BGC0000575/index.html#r1c1) |  | 15.0 | RiPP | lariatin | *Rhodococcus jostii* |
|  | [BGC0000571.1](https://mibig.secondarymetabolites.org/repository/BGC0000571/index.html#r1c1) |  | 15.0 | RiPP | burhizin | *Paraburkholderia rhizoxinica HKI 454* |
|  | [BGC0001552.1](https://mibig.secondarymetabolites.org/repository/BGC0001552/index.html#r1c1) |  | 15.0 | RiPP | citrulassin F | *Streptomyces avermitilis MA-4680 = NBRC 14893* |
|  | [BGC0000561](https://mibig.secondarymetabolites.org/repository/BGC0000560/index.html#r1c1) |  | 15.0 | RiPP | subtilomycin | *Bacillus subtilis* |
|  | [BGC0000616.1](https://mibig.secondarymetabolites.org/repository/BGC0000616/index.html#r1c1) |  | 15.0 | RiPP | amylocyclicin | *Bacillus velezensis FZB42* |
|  | [BGC0001551](https://mibig.secondarymetabolites.org/repository/BGC0001550/index.html#r1c1) |  | 15.0 | RiPP | citrulassin D | *Streptomyces katrae* |
| contig 3.1 | [BGC0001241.1](https://mibig.secondarymetabolites.org/repository/BGC0001241/index.html#r1c1) | **Cyclic lactone autoinducer** | 14.0 | Terpene | ergotamine | Claviceps fusiformis |
|  | [BGC0001267.1](https://mibig.secondarymetabolites.org/repository/BGC0001267/index.html#r1c1) |  | 14.0 | Terpene | lysergic acid, elymoclavine | Claviceps fusiformis |
|  | [BGC0001917.1](https://mibig.secondarymetabolites.org/repository/BGC0001917/index.html#r1c1) |  | 14.0 | Polyketide | 5-alkyl-1,2,3,4-tetrahydroquinolines, streptoaminals | Streptomyces nigrescens |
|  | [BGC0001463.1](https://mibig.secondarymetabolites.org/repository/BGC0001463/index.html#r1c1) |  | 13.0 | Other | 2,3,4,5-tetrabromopyrrole | Pseudoalteromonas piscicida |
|  | [BGC0001464.1](https://mibig.secondarymetabolites.org/repository/BGC0001464/index.html#r1c1) |  | 13.0 | Other | tetrabromopyrrole | Pseudoalteromonas sp. PS5 |
|  | [BGC0000261](https://mibig.secondarymetabolites.org/repository/BGC0000260/index.html#r1c1) |  | 13.0 | Polyketide | prodigiosin | Hahella chejuensis KCTC 2396 |
|  | [BGC0000541](https://mibig.secondarymetabolites.org/repository/BGC0000540/index.html#r1c1) |  | 13.0 | RiPP | paenibacillin | Paenibacillus polymyxa OSY-DF |
|  | [BGC0001896.1](https://mibig.secondarymetabolites.org/repository/BGC0001896/index.html#r1c1) |  | 13.0 | Other | carbazomycin B | Streptomyces luteoverticillatus |
|  | [BGC0000811.1](https://mibig.secondarymetabolites.org/repository/BGC0000811/index.html#r1c1) |  | 12.0 | Alkaloid | fumigaclavine C | Aspergillus fumigatus Af293 |
| contig 3.2 | [BGC0000617.1](https://mibig.secondarymetabolites.org/repository/BGC0000617/index.html#r1c1) | **RiPP-like** | 2.0 | RiPP | coagulin | *Bacillus coagulans* |
|  | [BGC0000249.1](https://mibig.secondarymetabolites.org/repository/BGC0000249/index.html#r1c1) |  | 18.0 | Polyketide | nogalamycin | *Streptomyces nogalater* |
|  | [BGC0000243.1](https://mibig.secondarymetabolites.org/repository/BGC0000243/index.html#r1c1) |  | 18.0 | Polyketide | macrotetrolide | *Streptomyces griseus subsp. griseus* |
|  | [BGC0000484.1](https://mibig.secondarymetabolites.org/repository/BGC0000484/index.html#r1c1) |  | 16.0 | RiPP | glycocin F | *Lactobacillus plantarum* |
|  | [BGC0001931](https://mibig.secondarymetabolites.org/repository/BGC0001930/index.html#r1c1) |  | 16.0 | RiPP | pallidocin | *Aeribacillus pallidus* |
|  | [BGC0001862.1](https://mibig.secondarymetabolites.org/repository/BGC0001862/index.html#r1c1) |  | 16.0 | RiPP | geocillicin | *Aeribacillus pallidus* |
|  | [BGC0001861.1](https://mibig.secondarymetabolites.org/repository/BGC0001861/index.html#r1c1) |  | 16.0 | RiPP | bacillicin BAG2O | *Bacillus cereus BAG2O-1* |
|  | [BGC0001571.1](https://mibig.secondarymetabolites.org/repository/BGC0001571/index.html#r1c1) |  | 16.0 | Terpene | demethoxyviridin | *Nodulisporium sp.* |
|  | [BGC0001388.1](https://mibig.secondarymetabolites.org/repository/BGC0001388/index.html#r1c1) |  | 15.0 | RiPP | gassericin E | *Lactobacillus gasseri* |
|  | [BGC0000619.1](https://mibig.secondarymetabolites.org/repository/BGC0000619/index.html#r1c1) |  | 15.0 | RiPP | gassericin T | *Lactobacillus gasseri* |
| contig 4.1 | [BGC0001479.1](https://mibig.secondarymetabolites.org/repository/BGC0001479/index.html#r1c1) | **NRPS** | 32.0 | NRP | anabaenopeptin NZ857, nostamide A | *Nostoc punctiforme PCC 73102* |
|  | [BGC0001261.1](https://mibig.secondarymetabolites.org/repository/BGC0001261/index.html#r1c1) |  | 31.0 | NRP | AM-toxin | *Alternaria alternata* |
|  | [BGC0000901](https://mibig.secondarymetabolites.org/repository/BGC0000900/index.html#r1c1) |  | 31.0 | Other | ferrichrome | *Aspergillus oryzae* |
|  | [BGC0001825.1](https://mibig.secondarymetabolites.org/repository/BGC0001825/index.html#r1c1) |  | 3.0 | NRP | xenematide | *Xenorhabdus nematophila AN6/1* |
|  | [BGC0000317.1](https://mibig.secondarymetabolites.org/repository/BGC0000317/index.html#r1c1) |  | 3.0 | NRP | cephalosporin C | *Acremonium chrysogenum ATCC 11550* |
|  | [BGC0000404.1](https://mibig.secondarymetabolites.org/repository/BGC0000404/index.html#r1c1) |  | 29.0 | NRP | penicillin | *Penicillium chrysogenum* |
|  | [BGC0001517.1](https://mibig.secondarymetabolites.org/repository/BGC0001517/index.html#r1c1) |  | 28.0 | NRP | asperphenamate | *Aspergillus terreus NIH2624* |
|  | [BGC0001132.1](https://mibig.secondarymetabolites.org/repository/BGC0001132/index.html#r1c1) |  | 28.0 | NRP | xenotetrapeptide | *Xenorhabdus nematophila ATCC 19061* |
|  | [BGC0001671.1](https://mibig.secondarymetabolites.org/repository/BGC0001671/index.html#r1c1) |  | 28.0 | NRP | monobactam | *Agrobacterium tumefaciens* |
|  | [BGC0000293.1](https://mibig.secondarymetabolites.org/repository/BGC0000293/index.html#r1c1) |  | 28.0 | NRP | acetylaszonalenin | *Aspergillus fischeri NRRL 181* |
| contig 9.1 | [BGC0000286.1](https://mibig.secondarymetabolites.org/repository/BGC0000286/index.html#r1c1) | **T3PKS** | 22.0 | Polyketide | viguiepinol | *Streptomyces sp. KO-3988* |
|  | [BGC0000554.1](https://mibig.secondarymetabolites.org/repository/BGC0000554/index.html#r1c1) |  | 16.0 | RiPP | SRO15-3108 | *Streptomyces filamentosus NRRL 15998* |
|  | [BGC0001551.1](https://mibig.secondarymetabolites.org/repository/BGC0001551/index.html#r1c1) |  | 16.0 | RiPP | citrulassin E | *Streptomyces glaucescens* |
|  | [BGC0000386.1](https://mibig.secondarymetabolites.org/repository/BGC0000386/index.html#r1c1) |  | 16.0 | NRP | malleobactin A, malleobactin B, malleobactin C, malleobactin D | *Burkholderia thailandensis E264* |
|  | [BGC0000551.1](https://mibig.secondarymetabolites.org/repository/BGC0000551/index.html#r1c1) |  | 16.0 | RiPP | SapB | *Streptomyces coelicolor A3(2)* |
|  | [BGC0000205.1](https://mibig.secondarymetabolites.org/repository/BGC0000205/index.html#r1c1) |  | 16.0 | Polyketide | bryostatin | *Candidatus Endobugula sertula* |
|  | [BGC0000504.1](https://mibig.secondarymetabolites.org/repository/BGC0000504/index.html#r1c1) |  | 16.0 | RiPP | cytolysin ClyLl, cytolysin ClyLs | *Plasmid pAD1* |
|  | [BGC0000489.1](https://mibig.secondarymetabolites.org/repository/BGC0000489/index.html#r1c1) |  | 16.0 | RiPP | enterocin AS-48 | *Enterococcus faecalis* |
|  | [BGC0000491](https://mibig.secondarymetabolites.org/repository/BGC0000490/index.html#r1c1) |  | 16.0 | RiPP | garvicin ML | *Lactococcus garvieae DCC43* |
|  | [BGC0000501](https://mibig.secondarymetabolites.org/repository/BGC0000500/index.html#r1c1) |  | 16.0 | RiPP | carnolysin A1, carnolysin A2 | *Carnobacterium maltaromaticum* |
| contig 15.1 | [BGC0000623.1](https://mibig.secondarymetabolites.org/repository/BGC0000623/index.html#r1c1) | **lanthipeptide-Class-II** | 99.0 | RiPP | plantaricin W α, plantaricin W β | *Lactobacillus plantarum* |
|  | [BGC0000554.1](https://mibig.secondarymetabolites.org/repository/BGC0000554/index.html#r1c1) |  | 45.0 | RiPP | SRO15-3108 | *Streptomyces filamentosus NRRL 15998* |
|  | [BGC0000516.1](https://mibig.secondarymetabolites.org/repository/BGC0000516/index.html#r1c1) |  | 35.0 | RiPP | geobacillin II | *Geobacillus thermodenitrificans* |
|  | [BGC0000504.1](https://mibig.secondarymetabolites.org/repository/BGC0000504/index.html#r1c1) |  | 35.0 | RiPP | cytolysin ClyLl, cytolysin ClyLs | *Plasmid pAD1* |
|  | [BGC0000617.1](https://mibig.secondarymetabolites.org/repository/BGC0000617/index.html#r1c1) |  | 33.0 | RiPP | coagulin | *Bacillus coagulans* |
|  | [BGC0000501](https://mibig.secondarymetabolites.org/repository/BGC0000500/index.html#r1c1) |  | 32.0 | RiPP | carnolysin A1, carnolysin A2 | *Carnobacterium maltaromaticum* |
|  | [BGC0001229.1](https://mibig.secondarymetabolites.org/repository/BGC0001229/index.html#r1c1) |  | 32.0 | RiPP | SBI-06990 A1, SBI-06989 A2 | *Streptomyces bingchenggensis BCW-1* |
|  | [BGC0001311.1](https://mibig.secondarymetabolites.org/repository/BGC0001311/index.html#r1c1) |  | 29.0 | RiPP | flavecins | *Ruminococcus flavefaciens FD-1* |
|  | [BGC0000552.1](https://mibig.secondarymetabolites.org/repository/BGC0000552/index.html#r1c1) |  | 29.0 | RiPP | SmbA, SmbB | *Streptococcus mutans* |
|  | [BGC0001407.1](https://mibig.secondarymetabolites.org/repository/BGC0001407/index.html#r1c1) |  | 28.0 | RiPP | bicereucin | *Bacillus cereus SJ1* |
| contig 22.1 | [BGC0000647.1](https://mibig.secondarymetabolites.org/repository/BGC0000647/index.html#r1c1) | **terpene** | 45.0 | Terpene | carotenoid | *Rhodobacter sphaeroides* |
|  | [BGC0000648.1](https://mibig.secondarymetabolites.org/repository/BGC0000648/index.html#r1c1) |  | 23.0 | Terpene | carotenoid | *Myxococcus xanthus* |
|  | [BGC0000656.1](https://mibig.secondarymetabolites.org/repository/BGC0000656/index.html#r1c1) |  | 23.0 | Terpene | zeaxanthin | *Xanthobacter autotrophicus Py2* |
|  | [BGC0000637.1](https://mibig.secondarymetabolites.org/repository/BGC0000637/index.html#r1c1) |  | 23.0 | Terpene | carotenoid | *Corynebacterium glutamicum* |
|  | [BGC0001227.1](https://mibig.secondarymetabolites.org/repository/BGC0001227/index.html#r1c1) |  | 21.0 | Terpene | isorenieratene | *Streptomyces collinus Tu 365* |
|  | [BGC0000633.1](https://mibig.secondarymetabolites.org/repository/BGC0000633/index.html#r1c1) |  | 21.0 | Terpene | carotenoid | *Streptomyces avermitilis* |
|  | [BGC0000641](https://mibig.secondarymetabolites.org/repository/BGC0000640/index.html#r1c1) |  | 2.0 | Terpene | carotenoid | *Enterobacteriaceae bacterium DC404* |
|  | [BGC0000645.1](https://mibig.secondarymetabolites.org/repository/BGC0000645/index.html#r1c1) |  | 2.0 | Terpene | carotenoid | *Halobacillus halophilus DSM 2266* |
|  | [BGC0000631](https://mibig.secondarymetabolites.org/repository/BGC0000630/index.html#r1c1) |  | 2.0 | Terpene | (2R,3S,3'S)-2-hydroxyastaxanthin | *Paracoccus haeundaensis* |
|  | [BGC0000646.1](https://mibig.secondarymetabolites.org/repository/BGC0000646/index.html#r1c1) |  | 19.0 | Terpene | β-carotein | Uncultured bacterium |
