# Supplementary material for: Bioprospecting of Ribosomally Synthesized and Post-translationally Modified Peptides Through Genome Characterization of a Novel Probiotic Lactiplantibacillus plantarum UTNGt21A Strain: A Promising Natural Antimicrobials Factory
Source: Front Microbiol. 2022 Apr 6;13:868025. doi: 10.3389/fmicb.2022.868025 (PMC9020862; doi:10.3389/fmicb.2022.868025)
Supplement: Supplementary file 1 [file Data_Sheet_1.zip › Table 2.DOCX]

**Supplementary Table S2.** Mapping overall results

| Library name | Total reads | Mapped reads | Coverage (%) | Depth | Ins. size (Std.) |
| --- | --- | --- | --- | --- | --- |
| UTNGt21A | 5,682,582 | 5,667,019 (99.73%) | 100.00 | 158.00 | 454.99 (95.24) |

Library name: Sample’s library name

Total reads: Total number of reads

Mapped reads: Total number of mapped reads

Coverage (%): The percentage of mapped sited (>= 1x)

Depth: Average mapping depth

Ins. size (Std.): The length between adapters and standard deviation of predicted length
