# Supplementary material for: Bioprospecting of Ribosomally Synthesized and Post-translationally Modified Peptides Through Genome Characterization of a Novel Probiotic Lactiplantibacillus plantarum UTNGt21A Strain: A Promising Natural Antimicrobials Factory
Source: Front Microbiol. 2022 Apr 6;13:868025. doi: 10.3389/fmicb.2022.868025 (PMC9020862; doi:10.3389/fmicb.2022.868025)
Supplement: Supplementary file 1 [file Data_Sheet_1.zip › Table 3.DOCX]

**Supplementary Table 3.** BUSCO analysis result

Used Lineage: bacteria_odb9 (number of species: 3663, number of BUSCOs: 148)

| Status | # BUSCOs | Percentage (%) |
| --- | --- | --- |
| Complete BUSCOs (C) |  | |
| Complete and single-copy BUSCOs (S) | 142 | 95.95 |
| Complete and duplicated BUSCOs (D) | 2 | 1.35 |
| Fragmented BUSCOs (F) | 1 | 0.68 |
| Missing BUSCOs (M) | 3 | 2.03 |
| Total BUSCO groups searched | 148 | 100 |

Status: A quantitative assessment list of the completeness in terms of expected gene content.

The following two conditions are used to create a status:

a. Expected range of scores

b. Expected range of length alignments

If both conditions are met, it is classified as Complete (These complete BUSCO matches are

either single-copy or duplicated). If length alignments are not met, it is classified as Fragmented.

If both conditions are not met, it is classified as Missing.

# BUSCOs: Identified count in sample

Percentage %: Identified percentage in sample
