# Supplementary material for: Bioprospecting of Ribosomally Synthesized and Post-translationally Modified Peptides Through Genome Characterization of a Novel Probiotic Lactiplantibacillus plantarum UTNGt21A Strain: A Promising Natural Antimicrobials Factory
Source: Front Microbiol. 2022 Apr 6;13:868025. doi: 10.3389/fmicb.2022.868025 (PMC9020862; doi:10.3389/fmicb.2022.868025)
Supplement: Supplementary file 1 [file Data_Sheet_1.zip › Table 5.DOCX]

**Supplementary Table 5.** Type strains used for the present dataset

| **Kind** | **Species cluster** | **Subspecies cluster** | **Preferred name** | **Deposit** | **Base pairs** | **Percent G+C** | **No. proteins** |
| --- | --- | --- | --- | --- | --- | --- | --- |
| type strain | 1 | 0 | *Lactiplantibacillus pingfangensis* | NCIMB 15187 | 2,889,170 | 44.16 | 2677 |
| type strain | 2 | 1 | *Lactiplantibacillus daoliensis* | NCIMB 15181 | 2,627,884 | 43.66 | 2418 |
| type strain | 3 | 2 | *Lactiplantibacillus nangangensis* | NCIMB 15186 | 2,894,027 | 44.31 | 2759 |
| type strain | 4 | 3 | *Lactiplantibacillus herbarum* | TCF032-E4 | 2,896,305 | 43.5 | 2807 |
| type strain | 5 | 4 | *Lactiplantibacillus fabifermentans* | DSM 21115 | 3,278,256 | 45.04 | 2973 |
| type strain | 6 | 5 | *Lactiplantibacillus argentoratensis* | DSM 16365 | 3,185,634 | 45.02 | 2940 |
| type strain | 7 | 6 | *Lactiplantibacillus pentosus* | DSM 20314 | 3,629,620 | 46.31 | 3276 |
| type strain | 8 | 7 | *Lactiplantibacillus paraplantarum* | DSM 10667 | 3,394,677 | 43.69 | 3193 |
| type strain | 9 | 8 | *Lactiplantibacillus xiangfangensis* | LMG 26013 | 2,988,776 | 45.1 | 2758 |
| type strain | 10 | 9 | *Lactobacillus arizonensis* | DSM 13273 | 3,438,114 | 44.24 | 3254 |
| type strain | 10 | 9 | *Lactiplantibacillus plantarum* | DSM 20174 | 3,250,154 | 44.5 | 2980 |
| type strain | 10 | 9 | *Lactiplantibacillus plantarum* | ATCC 14917 | 3,198,761 | 44.48 | 3154 |
| user strain | 10 | 9 | Gt21A contigs | UTNGt21A | 3,558,611 | 43.96 | 3471 |
