# Supplementary material for: Bioprospecting of Ribosomally Synthesized and Post-translationally Modified Peptides Through Genome Characterization of a Novel Probiotic Lactiplantibacillus plantarum UTNGt21A Strain: A Promising Natural Antimicrobials Factory
Source: Front Microbiol. 2022 Apr 6;13:868025. doi: 10.3389/fmicb.2022.868025 (PMC9020862; doi:10.3389/fmicb.2022.868025)
Supplement: Supplementary file 1 [file Data_Sheet_1.zip › Table 6.DOCX]

**Supplementary Table 6**. Predicted prophage regions within the UTNGt21A genome.

| **Contig** | **Region** | **Region length (kb)** | **Completeness** | **Score** | **# Total proteins** | **Region position** | **Most common Phage** | **GC%** |
| --- | --- | --- | --- | --- | --- | --- | --- | --- |
| 4 | 1 | 44.3 | intact | 150 | 68 | 116832-161151 | PHAGE_Lactob_phig1e_NC_004305(11) | 41.22 |
| 5 | 2 | 14.7 | incomplete | 30 | 10 | 184007-198739 | PHAGE_Staphy_AJ_2017_NC_048644(1) | 40.56 |
| 7 | 3 | 47.8 | intact | 150 | 63 | 136569- 184377 | PHAGE_Lactob_Sha1_NC_019489(10) | 40.84 |
| 10 | 4 | 38.7 | intact | 140 | 54 | 131152-169879 | PHAGE_Lactob_Sha1_NC_019489(27) | 40.78 |
| 12 | 5 | 9.1 | incomplete | 40 | 15 | 546  -  9715 | PHAGE_Lactob_Sha1_NC_019489(2) | 41.23 |
| 15 | 6 | 5.6 | incomplete | 60 | 10 | 48903  -  54597 | PHAGE_Lactob_phiAT3_NC_005893(2) | 39.03 |
| 19 | 7 | 17.5 | incomplete | 40 | 38 | 1  -  17563 | PHAGE_Lactob_Sha1_NC_019489(13) | 38.47 |
| 22 | 8 | 19.2 | incomplete | 50 | 37 | 1  -  19222 | PHAGE_Lactob_Sha1_NC_019489(11) | 39.02 |
| 24 | 9 | 39.6 | intact | 130 | 57 | 1  -  39628 | PHAGE_Lister_B025_NC_009812(9) | 40.86 |

Legend: region: the number assigned to the region; region length: the length of the sequence in that region; completeness: a prediction of whether the region contains a intact or incomplete prophage based on the criteria: intact (score > 90%); questionable (score 70-90); incomplete (score < 70%); score: the score of the region based on the mentioned criteria; # total proteins: the number of ORFs present in the region; Region position: the start and end positions of the region on the bacterial chromosome; Most common phage: the phages (s) with the highest number of proteins most similar to those in the region; GC%: the percentage of GC nucleotides of the region
