# Supplementary material for: Bioprospecting of Ribosomally Synthesized and Post-translationally Modified Peptides Through Genome Characterization of a Novel Probiotic Lactiplantibacillus plantarum UTNGt21A Strain: A Promising Natural Antimicrobials Factory
Source: Front Microbiol. 2022 Apr 6;13:868025. doi: 10.3389/fmicb.2022.868025 (PMC9020862; doi:10.3389/fmicb.2022.868025)
Supplement: Supplementary file 1 [file Data_Sheet_1.zip › Table 7.DOCX]

**Supplementary Table 7.** Antibiotic susceptibility of the UTNGt21A strain

| **Antimicrobial E-test strip** | **MIC (mg/L)** | **Susceptibility*** | **EFSA cut-off values (mg/L) *Lactobacillus* obligate**  **Heterofermentative [29]** |
| --- | --- | --- | --- |
| Amoxicillin | 1.5 | S | 4 |
| Amoxicillin: clavulanic acid | 0.016 | S | 4 |
| Ampicillin | 0.5 | S | 2 |
| Cefotaxime | 2 | S | 16 |
| Erythromycin | 1 | S | 1 |
| Gentamycin | 16 | S | 16 |
| Penicillin | 0.25 | S | 0.25 |
| Tetracycline | 32 | S | 32^**^ |

*The microbiological breakpoints reported by the FEEDAP were used to categorize lactobacilli as susceptible or resistant [29]. The strains showing a MIC higher than the EFSA breakpoint were considered resistant. Susceptible (S): a bacterial strain is defined as susceptible when it is inhibited at a concentration of a specific antimicrobial equal to or lower than the established cut-off value (S ≤ x mg / L). Resistant (R): a bacterial strain is defined as resistant when it is not inhibited at a concentration of a specific antimicrobial above the established cut-off value (R> x mg / L). ** MIC reference for *L. plantarum.*
