# Supplementary material for: Bioprospecting of Ribosomally Synthesized and Post-translationally Modified Peptides Through Genome Characterization of a Novel Probiotic Lactiplantibacillus plantarum UTNGt21A Strain: A Promising Natural Antimicrobials Factory
Source: Front Microbiol. 2022 Apr 6;13:868025. doi: 10.3389/fmicb.2022.868025 (PMC9020862; doi:10.3389/fmicb.2022.868025)
Supplement: Supplementary file 1 [file Data_Sheet_1.zip › Table 8.DOCX]

**Supplementary Table 8.** The list of VFDB gene ID predicted within the UTNGt21A genome

| **Gene ID** | **% Identity** | **VFDB gene ID** |
| --- | --- | --- |
| gene00008 | 48.9 | VFG031747(gi:406032839) (regX3) Sensory transduction protein regX3 |
| gene00013 | 52.3 | VFG005533(gi:24380491) (htrA/degP) serine protease HtrA |
| gene00121 | 46.8 | VFG030724(gi:433649467) (sugC) carbohydrate ABC transporter ATP-binding protein, CUT1 family |
| gene00172 | 51.1 | VFG031457(gi:15609582) (ndk) Probable nucleoside diphosphate kinase NdkA (NDK) (NDP kinase) (nucleoside-2-P kinase) |
| gene00188 | 42.2 | # |
| gene00202 | 44.4 | VFG024189(gi:387874359) (mprA) two component response transcriptional regulatory protein MprA |
| gene00208 | 44.8 | VFG016389(gi:42784428) (lytR) membrane-bound transcriptional regulator LytR |
| gene00235 | 40.4 | VFG030679(gi:118473047) (sugC) ABC transporter ATP-binding protein |
| gene00244 | 53 | VFG006719(gi:16800743) (lap) hypothetical protein |
| gene00257 | 40.5 | # |
| gene00258 | 50.3 | # |
| gene00280 | 41.3 | VFG030696(gi:387874659) (sugC) ABC transporter, ATP-binding protein SugC |
| gene00295 | 41.1 | # |
| gene00393 | 44 | VFG047710 (FN3523_0021) carbamoyl-phosphate synthase large chain |
| gene00399 | 44.8 | VFG015903(gi:71735587) (argK) phaseolotoxin-insensitive ornithine carbamoyltransferase |
| gene00461 | 40 | VFG036559(gi:313668880) (fbpC) iron-uptake permease ATP-binding protein |
| gene00547 | 45.3 | VFG013731(gi:68536864) (fagC) putative iron ABC transport system, ATP-binding protein |
| gene00592 | 45.7 | VFG031404(gi:379747038) (ctpV) metal cation transporter p-type ATPase, CtpV |
| gene00754 | 48.4 | # |
| gene00760 | 50 | VFG037029(gi:59802086) (katA) catalase |
| gene00781 | 40.6 | VFG005069(gi:49482401) (cap8J) capsular polysaccharide synthesis enzyme |
| gene00798 | 67.9 | VFG032507(gi:424714946) (bsh) Choloylglycine hydrolase |
| gene00850 | 57.5 | VFG016308(gi:42784425) (galE) UDP-glucose 4-epimerase |
| gene00984 | 72 | VFG005582(gi:125717729) (eno) Enolase, putative |
| gene00987 | 58.8 | VFG005360(gi:24378857) (plr/gapA) glyceraldehyde-3-phosphate dehydrogenase |
| gene00990 | 70.4 | # |
| gene00999 | 41.4 | VFG018246(gi:59711152) (luxS) S-ribosylhomocysteinase |
| gene01013 | 75.7 | VFG005871(gi:94991482) (hasC) UTP--glucose-1-phosphate uridylyltransferase |
| gene01015 | 56.1 | VFG006806(gi:16804520) (lgt) hypothetical protein |
| gene01026 | 42.8 | VFG031738(gi:169631126) (regX3) Sensory transduction protein RegX3 |
| gene01035 | 49 | VFG005841(gi:24378755) (rgpG) putative glycosyl transferase N-acetylglucosaminyltransferase), RgpG |
| gene01037 | 67.9 | VFG012103(gi:125975373) (groEL) chaperonin GroEL |
| gene01052 | 57.5 | VFG016308(gi:42784425) (galE) UDP-glucose 4-epimerase |
| gene01176 | 57.5 | VFG008112(gi:507419452) (panD) aspartate 1-decarboxylase |
| gene01213 | 41.1 | VFG047736 (F7308_0022) carbamoyl-phosphate synthase small chain |
| gene01214 | 45 | VFG047713 (FNFX1_0026) hypothetical protein |
| gene01257 | 47.6 | VFG032200(gi:347548334) (lplA1) putative lipoate protein ligase A |
| gene01361 | 40.9 | VFG031747(gi:406032839) (regX3) Sensory transduction protein regX3 |
| gene01415 | 41 | VFG038840(gi:507521851) (flmH) 3-oxoacyl-ACP reductase |
| gene01417 | 42.6 | # |
| gene01419 | 42.5 | VFG011402(gi:23502030) (fabZ) (3R)-hydroxymyristoyl ACP dehydratase |
| gene01434 | 42.3 | VFG009364(gi:126435879) (trpD) anthranilate phosphoribosyltransferase |
| gene01454 | 41.5 | # |
| gene01461 | 40.1 | VFG046604 (Fphi_1467) ribulose-phosphate 3-epimerase |
| gene01464 | 46.7 | VFG032386(gi:347549217) (stp) putative phosphoprotein phosphatase |
| gene01478 | 55.3 | VFG012175(gi:126698804) (CD1208) putative RNA methyltransferase |
| gene01530 | 76.8 | VFG006826(gi:16803417) (lisR) two-component response regulator |
| gene01532 | 63.3 | VFG048851 (gnd) 6-phosphogluconate dehydrogenase |
| gene01590 | 43.5 | # |
| gene01608 | 45.1 | # |
| gene01621 | 44.9 | VFG013269(gi:33152420) (orfM) putative deoxyribonucleotide triphosphate pyrophosphatase |
| gene01654 | 40.8 | VFG036552(gi:385339601) (fbpC) iron(III) ABC transporter ATP-binding protein |
| gene01714 | 50.3 | VFG016424(gi:118480308) (manA) mannose-6-phosphate isomerase |
| gene01820 | 58 | # |
| gene01831 | 41.3 | VFG016423(gi:118480307) (lytR) transcription antiterminator |
| gene01962 | 40.1 | VFG033009(gi:424714165) (oatA) Putative peptidoglycan O-acetyltransferase YrhL |
| gene01979 | 43.2 | VFG043551(gi:15827894) (ML1683) histone-like protein |
| gene01988 | 44 | VFG043456(gi:22537734) (scpB) segregation and condensation protein B |
| gene01998 | 50.5 | VFG049195 (clpB) protein disaggregation chaperone |
| gene02005 | 46.4 | VFG007915(gi:126435440) (ddrA) daunorubicin resistance ABC transporter ATPase subunit |
| gene02014 | 50.8 | VFG005580(gi:15900994) (eno) phosphopyruvate hydratase |
| gene02049 | 60.7 | VFG026980(gi:386005579) (sigA/rpoV) RNA polymerase sigma factor |
| gene02083 | 49.4 | VFG032820(gi:289434243) (dltA) D-alanine-activating enzyme |
| gene02087 | 60.5 | VFG043573(gi:15605121) (CT396) molecular chaperone DnaK |
| gene02108 | 49.8 | VFG045683(gi:529234457) (EFAU085_01747) phosphatidate cytidylyltransferase |
| gene02109 | 69.7 | VFG045688(gi:383329042) (uppS) undecaprenyl diphosphate synthase |
| gene02126 | 41 | VFG012939(gi:110804318) (gtrB) bactoprenol glucosyl transferase |
| gene02194 | 42.6 | VFG016506(gi:71894295) (pdhB) pyruvate dehydrogenase E1 component, beta subunit |
| gene02222 | 71.2 | VFG046474 (OOM_0626) elongation factor |
| gene02223 | 52.2 | VFG005547(gi:25010179) (tig/ropA) trigger factor |
| gene02233 | 43.7 | VFG016421(gi:49478949) (BT9727_4960) tyrosine-protein kinase |
| gene02236 | 47.5 | VFG046630 (FN3523_1291) sugar transferase |
| gene02363 | 57.2 | VFG019127(gi:169834375) (SPH_0465) UDP-N-acetylglucosamine 2-epimerase |
| gene02376 | 77 | VFG005898(gi:125718225) (rmlA) Glucose-1-phosphate thymidylytransferase, putative |
| gene02377 | 46.7 | VFG048807 (rmlC) dTDP-4-dehydrorhamnose 3,5-epimerase |
| gene02378 | 79.6 | VFG006022(gi:116627988) (STER_1222) dTDP-D-glucose 4,6-dehydratase |
| gene02379 | 58.4 | VFG018667(gi:157149997) (rfbD) dTDP-4-dehydrorhamnose reductase |
| gene02390 | 62.5 | # |
| gene02399 | 48.7 | VFG005844(gi:116627835) (STER_1057) Polysaccharide Transporter, PST family |
| gene02400 | 61.3 | VFG016432(gi:118480316) (epsE) sugar transferase |
| gene02426 | 43.8 | VFG032255(gi:289435537) (oppA) oligopeptide ABC transporter substrate-binding protein |
| gene02430 | 49.2 | VFG016532(gi:42561491) (oppF) oligopeptide ABC transporter, permease component |
| gene02433 | 62.7 | # |
| gene02508 | 58.9 | VFG031940(gi:404413714) (lap) bifunctional aldehyde-alcohol dehydrogenase |
| gene02533 | 52.8 | VFG030724(gi:433649467) (sugC) carbohydrate ABC transporter ATP-binding protein, CUT1 family |
| gene02573 | 44.8 | VFG006777(gi:116872329) (lplA1) lipoyltransferase and lipoate-protein ligase family protein |
| gene02586 | 53.6 | VFG032847(gi:116873915) (gtcA) cell wall teichoic acid glycosylation protein |
| gene02668 | 44.4 | VFG036559(gi:313668880) (fbpC) iron-uptake permease ATP-binding protein |
| gene02690 | 42.1 | VFG032493(gi:347549240) (lspA) putative signal peptidase II |
| gene02705 | 48.1 | VFG005195(gi:116516285) (pavA) adherence and virulence protein A |
| gene02725 | 50.7 | VFG006042(gi:76788234) (cpsY) transcriptional regulator CpsY |
| gene02820 | 46.8 | VFG030686(gi:169628466) (sugC) Probable sugar ABC transporter, ATP-binding protein SugC |
| gene02926 | 50.3 | VFG015885(gi:71736839) (cysC1) adenylylsulfate kinase |
| gene03035 | 48.7 | VFG049038 (KPN2242_15480) hypothetical protein |
| gene03038 | 50.1 | VFG005582(gi:125717729) (eno) Enolase, putative |
| gene03089 | 44.1 | VFG042130(gi:13475288) (mlr6326) putative DNA invertase |
| gene03115 | 44.3 | VFG013515(gi:148826007) (mrsA/glmM) predicted phosphomannomutase |
| gene03202 | 41.9 | VFG016229(gi:30020330) (hlyIII) Hemolysin III |
| gene03234 | 50.8 | VFG019048(gi:76787756) (psaA) metal ABC transporter, metal-binding lipoprotein |
| gene03333 | 40.9 | VFG018243(gi:28899311) (luxS) S-ribosylhomocysteinase |

VFDB: virulence factor database, <http://www.mgc.ac.cn/VFs/main.htm>
