# Supplementary material for: Bioprospecting of Ribosomally Synthesized and Post-translationally Modified Peptides Through Genome Characterization of a Novel Probiotic Lactiplantibacillus plantarum UTNGt21A Strain: A Promising Natural Antimicrobials Factory
Source: Front Microbiol. 2022 Apr 6;13:868025. doi: 10.3389/fmicb.2022.868025 (PMC9020862; doi:10.3389/fmicb.2022.868025)
Supplement: Supplementary file 1 [file Data_Sheet_1.zip › Table 9.DOCX]

**Supplementary Table 9.** Putative bacteriocins annotated within the UTNGt21A genome and the percentage identity with the reference genome (*L. plantarum* WCFS1).

| **Genes (locus tag)** | **Product** | **Description** | **Library name. No. of genes / % identity with the reference genome (WCFS1)** | |  |
| --- | --- | --- | --- | --- | --- |
|  |  |  | **UTNGt21A** | |  |
|  |  |  |  |  |  |
| *lcnD* | Lactococcin A secretion protein LcnD | Transport protein ComB | 1 | 30.837 |  |
| *lagD (lagD_1)* | Lactococcin-G-processing and transport ATP-binding protein LagD | (ABC) transporter | 1 | 60.815 |  |
| *lagD_2* | Lactococcin-G-processing and transport ATP-binding protein LagD | (ABC) transporter | 1 | 45.421 |  |
| *lagD_3* | Lactococcin-G-processing and transport ATP-binding protein LagD | (ABC) transporter | 1 | 38.788 |  |
| *lagD_4* | Lactococcin-G-processing and transport ATP-binding protein LagD | (ABC) transporter | 1 | 32.959 |  |
| *lagD_5* | Lactococcin-G-processing and transport ATP-binding protein LagD | (ABC) transporter | 1 | 43.515 |  |
| *nisP* | Nisin leader peptide-processing serine protease NisP | peptidase (S8 and S53, subtilisin, kexin, sedolisin) | 1 | 33.923 |  |
| G21A_02824 | hypothetical protein | Lanthionine synthetase C family protein | 1 | 25.000 |  |
| G21A_02825 | hypothetical protein | Lanthionine synthetase C family protein | 1 | 24.462 |  |
| G21A_00323 | hypothetical protein | CAAX amino terminal protease family | 1 | 39.286 |  |
| G21A_02838 | hypothetical protein | Transport protein ComB | 1 | 37.931 |  |
| *bdb* | Disulfide bond formation protein | Bacteriocin transport accessory protein | 1 | 32.673 |  |
| G21A_00737 | hypothetical protein | enterolysin_A | 1 | 40.940 |  |
| G21A_00730 | hypothetical protein | Bacteriocin production related histidine kinase | 1 | 35.206 |  |
| G21A_00305 | hypothetical protein | Bacteriocin class II with double-glycine leader peptide; | 1 | (-) |  |

(-) no identity
